# Gestational diabetes mellitus – more than the eye can see – a warning sign for future maternal health with transgenerational impact

**DOI:** 10.3389/fcdhc.2025.1527076

**Published:** 2025-04-01

**Authors:** Manal Massalha, Rula Iskander, Haya Hassan, Etty Spiegel, Offer Erez, Zohar Nachum

**Affiliations:** ^1^ Department of Obstetrics and Gynecology, Emek Medical Center, Afula, Israel; ^2^ Rappaport Faculty of Medicine, Technion, Institute of technology, Haifa, Israel; ^3^ Department of Obstetrics and Gynecology, Soroka University Medical Center, Beer Sheva, Israel; ^4^ Faculty of Medicine, Ben Gurion University of the Negev, Beer Sheva, Israel; ^5^ Department of Obstetrics and Gynecology, Hutzel Women’s Hospital, Wayne State University, Detroit, MI, United States

**Keywords:** metabolic syndrome, insulin, preeclampsia, pregnancy complications, lipid profile, macrosomia

## Abstract

Gestational diabetes mellitus (GDM) is regarded by many as maternal maladaptation to physiological insulin resistance during the second half of pregnancy. However, recent evidence indicates that alterations in carbohydrate metabolism can already be detected in early pregnancy. This observation, the increasing prevalence of GDM, and the significant short and long-term implications for the mother and offspring call for reevaluation of the conceptual paradigm of GDM as a syndrome. This review will present evidence for the syndromic nature of GDM and the controversies regarding screening, diagnosis, management, and treatment.

## Gestational diabetes as an obstetric syndrome

Maternal insulin resistance, which develops physiologically during the second half of pregnancy, serves the increasing fetal requirements for free glucose required to support growth. Disruption in the maternal metabolic support of pregnancy can lead to pregnancy complications such as fetal growth restriction, and preterm birth in case of in sufficient support (1) and diabetes mellitus on cases of increased glucose supply. However, GDM is an obstetric syndrome characterized by multiple etiologies, a long subclinical phase, fetal involvement, and complex gene-environment interactions (2).

During the subclinical phase, elevated amniotic fluid insulin concentrations in the mid-trimester (OR 1.9, 95% CI 1.3 - 2.4) (3), the maternal metabolic profile during the first trimester (4–7), and the proteomics profile in the first trimester (8–12) are all associated with increased risk of GDM.

The fetus may also play a part in the development of GDM as maternal insulin resistance develops in response to placental hormones, specifically human placental lactogen (hPL). Conversely, the fetus is affected *in utero* by the high-glucose environment leading to macrosomia, polyhydramnios, and neonatal hypoglycemia (1).

A genetic predisposition for GDM has been proposed involving post-receptor insulin signaling and downregulation of insulin receptor substrate-1 contributing to a reduction in glucose uptake by skeletal muscle. Furthermore, a systematic review identified 9 single nucleotide polymorphisms (SNPs) associated with and increased risk of GDM suggesting that gene-environment interactions affect a patient’s risk (13).

## Changes in the epidemiology of gestational diabetes

**Figure 1:**
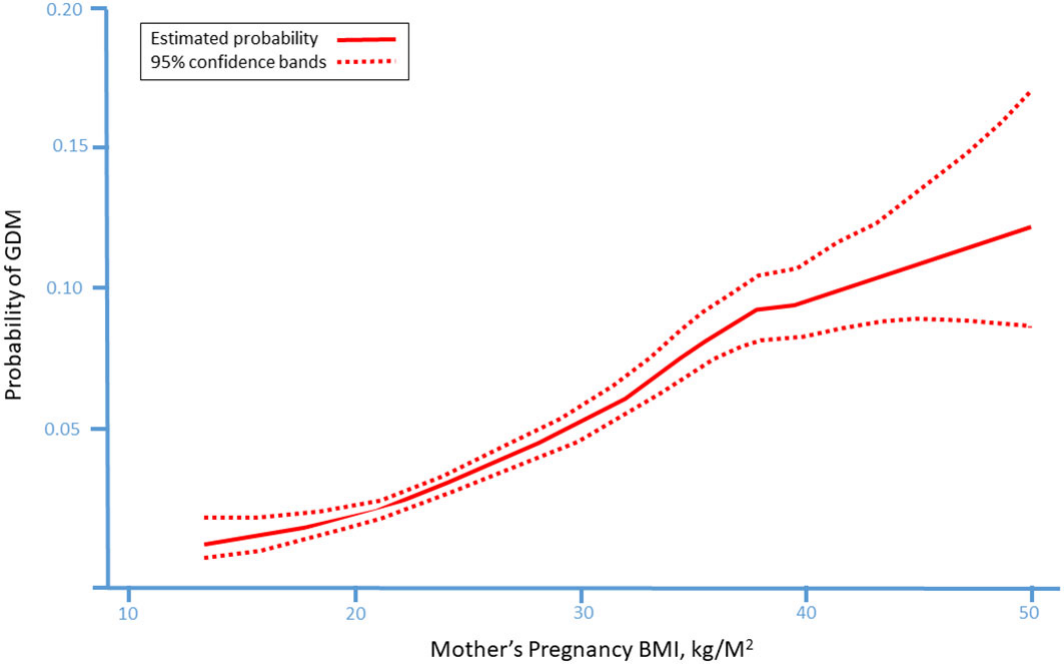
The probability of gestational diabetes mellitus (GDM) in according to mother’s pre-pregnancy body mass index (BMI) presenting a linear association between pre-pregnancy BMI and the probability to develop GDM during pregnancy. [23 with permission].

The prevalence of GDM in a cohort of over 100 million deliveries in the US increased 23-fold from 1979 until 2010 (14) and is still rising, reaching 9.2% in some studies (15). This is a global problem, with studies in Australia, North Africa and the Middle East confirming the trend and with a prevalence as high as 36% (16). The rate of GDM is influenced by factors such as ethnicity, maternal age, and obesity (17–22). Indeed, the rate of GDM in overweight, obese, and morbid obese women, reaches 10%, 15% and 21% respectively (23) (**Figure 1**).

## Underlying mechanisms of gestational diabetes

**Figure 2:**
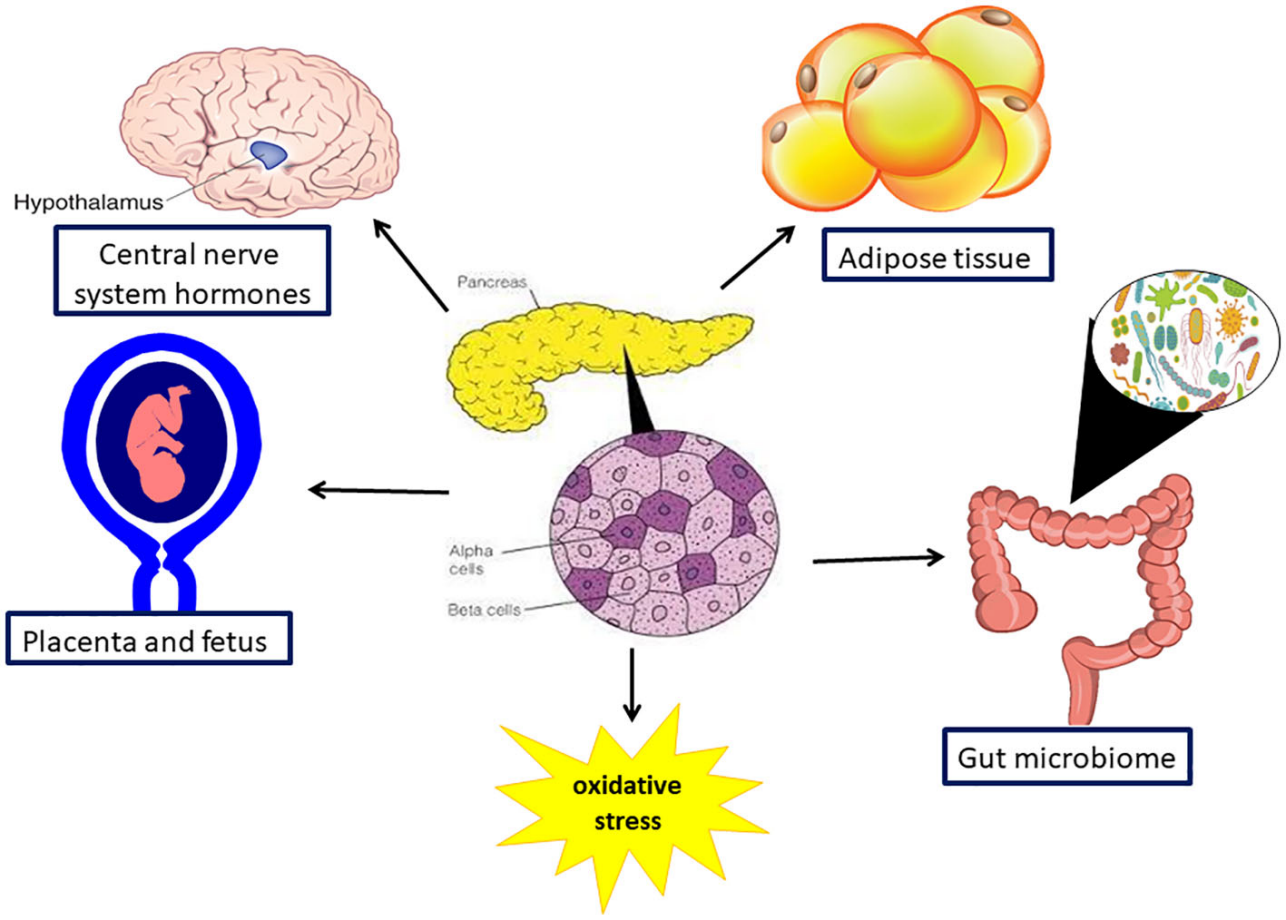
Organs involved in pathophysiology in gestation diabetes mellitus. Gestational diabetes is a multisystem disorder as it affect the pancreas, gut and its microbiome, the brain and the placenta and the fetus.

Significant modifications occur in maternal metabolism during pregnancy, which are a prerequisite to adapting maternal and fetal nutritional requirements throughout gestation (**Figure 2**). The most prominent maternal metabolic adaptation during pregnancy is the change in insulin sensitivity, which depends on the metabolic requirements of the developing fetus. During the early stages of pregnancy insulin sensitivity increases in preparation for the increasing insulin demand in later gestation, encouraging uptake of glucose into adipose stores (24). Conversely, as pregnancy advances, increasing maternal and placental hormones, including estrogen, progesterone, cortisol, leptin, hPL, and placental growth hormone (PlGH) simultaneously promote a state of insulin resistance (25). Consequently, blood glucose is elevated and its active transport across the placenta contributes to the accelerated fetal growth during the third trimester. Hence, the maternal pancreas continues to increase insulin production and secretion, to prevent hyperglycemia. When compensation is only partially functional, due to lack of sufficient insulin reserves, maternal hyperglycemia and GDM ensue (26).

The most prominent of the multi-organ molecular processes associated with the development of GDM is pancreatic β-cell dysfunction. This is defined as the loss of ability to sense circulating glucose concentrations or to release sufficient insulin to maintain euglycemia, resulting from prolonged excessive insulin secretion or post-receptor signaling defects. Consequently, most of the reported susceptibility genes are related to β-call function (27) (**Table 1**) and the metabolic stress of pregnancy may uncover minor perturbations in previously healthy women, or lead to uncontrolled hyperglycemia and severe cellular dysfunction in cases of pre-existing β-cell dysfunction, a process called glucotoxicity (28). GDM has also been associated with depletion of β-cell mass or number (29, 30) in animal models and post-mortems.

**Table 1:** Candidate genes demonstrating association with GDM in meta-analysis.

Gene	Chromosome	Encoded protein	Protein function
IRS1	2	Insulin receptor substrate 1	Substrate of insulin receptor tyrosine kinase; key molecule in the insulin signaling pathway
IGF2BP2	3	Insulin-like growth factor 2 mRNA-binding protein 2	Binds insulin-like growth factor 2- mRNA and may regulate protein translation; risk allele associated with decreased insulin secretion
CDKAL1	6	CDK5 regulatory subunit associated protein 1 like-1	A tRNA methylthiotransferase; non-pregnant carriers of the risk alleles have impaired oral and intravenous glucose stimulated insulin secretion
GCK	7	Glucokinase	Phosphorylates glucose in pancreatic β-cells and hepatocytes; involved in the regulation of insulin secretion
TCF7L2	10	Transcription factor 7-like 2	Transcription factor and member of the Wnt signaling pathway; risk allele associated with reduced insulin secretion
MTNR1B	11	Melatonin receptor 1B	G-protein coupled receptor that is expressed on β-cells, binds melatonin and may antagonize insulin release
KCNJ11	11	Potassium inwardly rectifying channel, subfamily J, member 11	Integral membrane protein and inward-rectifier type potassium channel which is controlled by G-proteins and associated with the sulfonylurea receptor; involved in the regulation of insulin secretion
KCNQ1	11	Potassium voltage-gated channel, KQT-like subfamily, member 1	Voltage-gated potassium channel; involved in the regulation of insulin secretion

Adapted with permission from: Lowe WL, Jr., Karban J. Genetics, genomics and metabolomics: new insights into maternal metabolism during pregnancy. Diabet Med 2014; (31:254-262) (27).

Chronic insulin resistance has also been implicated in the development of GDM as demonstrated by a 54% reduction in insulin stimulated glucose uptake compared to normal pregnancy. This is mostly due to a reduced inter-cellular response to insulin and alterations in downstream signaling (31–33), which is also affected by saturated fatty acids, pro-inflammatory cytokines, and adiponectin (34).

Neuro-hormonal networks are critical in the regulation of appetite, energy expenditure and basal metabolic rate, which are mediated by adipocytokines such as leptin and adiponectin. Leptin is associated with maternal obesity and pre-pregnancy body mass index (BMI), but placental production of leptin is notably increased in women with GDM, contributing to hyperleptinemia and maternal insulin resistance as well as fetal macrosomia (35–40). Adiponectin has the inverse action of leptin, and its plasma concentrations decrease as the adipose tissue mass increases, which is also associated with GDM (41, 42). Hypertrophy of adipose tissue and down-regulation of regulators for insulin signaling transporters of fatty acids lead to glucotoxicity and lipotoxicity culminating in fat accumulation in the muscles and liver. Additionally, excessive fat contributes to maternal systemic inflammation by the secretion of pro inflammatory cytokines (43–51).

Oxidative stress is also an important factor; women with GDM have over production of free radicals and their scavenging capability is decreased. The free radicals of reactive oxygen species affect glucose uptake by cells and glycogen synthesis in the liver and muscles, further contributing to the metabolic imbalance associating with gestational diabetes (52–54).

The placenta is both a major contributor and a target organ of GDM. Placental hormones like hPL mediate maternal insulin resistance. During GDM the placenta is affected as the high concentration of insulin in these patients increases placental glucose uptake and contributes to excessive fetal growth and macrosomia (55, 56). Similar effect is observed in placental amino acid transport that is markedly increased further enhancing fetal growth (57). Interestingly, it is lipids placental transport that is affected in GDM. Indeed, 67% of lipid pathways placental gene expressions are affected in GDM versus only 9% of placental genes involved in glucose pathways. These observations may also explain the link between fetal macrosomia and maternal obesity (58).

## Microbiome and gestational diabetes

Pregnancy is associated with fundamental changes in the gut microbiota in comparison to the non-pregnant state. These changes are evidence during the second and third trimesters, include a reduction in alpha diversity, and increase in the beta diversity of pregnant women in comparison to the non-pregnant state. These chances have been attributed to the physiological insulin resistance of pregnancy. Indeed, the chances in the gut microbiome during the second and third trimesters are similar to those observed in patients with metabolic syndrome (59–62).

The gut microbiome plays an essential role in carbohydrate and lipid metabolism, insulin homeostasis, and inflammation (63, 64). Ya-Shu Kuang et al, observed associations between gut microbiome and GDM status. Functional analysis showed a higher quantity of lipopolysaccharide, energy metabolism pathways, membrane transport, and phosphotransferase systems in the microbiome of GDM patients, while the microbiome of controls was enhanced in the amino acid metabolic pathways (65). Another prospective cohort study indicates that trace element exposure is associated with specific gut microbiome features that may contribute to GDM development (66). Recently published meta-analysis conducted the correlation between intestinal flora and GDM. It revealed a correlation between intestinal microecological changes and occurrence of GDM, which manifested as a decrease in the level of probiotics, an increase in the level of intestinal bacteria and other strains, along with the increase in the level of inflammatory factors in women with GDM (67).

Moreover, GDM affects not only the gut, but also the vaginal microbiome. Indeed, vaginal dysbiosis is more prevalent among women with GDM then in those with normal pregnancy. In addition, the vaginal microbiome of women with GDM had lower alpha diversity indices then those with the normal pregnancy (68, 69).

The consumption of probiotic supplements is widely investigated for their beneficial effects on the treatment of metabolic diseases (70). The World Health Organization (WHO) has defined the probiotics as live microorganisms that when taken properly contribute health benefits on the host (70–73). Intake of probiotics is safe and effective in regulating the human gut microbial composition and function, to support favorable metabolic activity, produce beneficial metabolites, and normalize the gut microbiota (71, 73, 74). In addition, supplementation with probiotics has been shown to improve glycemic control and lipid profile in patients with type 2 diabetes mellitus (74–77).

Probiotic supplementation, with Lactobacillus rhamnosus and Bifidobacteriumlactis, in non-obese women during pregnancy resulted in a decline in the rate of GDM from 34% to 13% according to Luoto et al. (76). However, Asemi et al. (77) did not find an impact on glycemic control in women with GDM supplemented with probiotics. Similar results were reported by Lindsay et al. that found that in women with GDM probiotic capsule intervention had no impact on glycemic control (76). Conversely, a few recent studies demonstrated a positive effect of different strains of probiotics on pregnant women with GDM and showed that probiotic supplement appeared to improve glucose metabolism and insulin resistance, as well as weight gain among those women (78, 79). Yefet et al. in a recently published meta-analysis of placebo-controlled RCTs (14 studies, 854 women), which evaluated the effects of probiotic supplements in GDM, found that probiotics had significantly lower mean fasting serum glucose, fasting serum insulin, insulin resistance, total and very-low-density lipoprotein cholesterol, and triglycerides levels. Decreased neonatal birth weight was observed in Lactobacillus acidophilus containing supplements (80). This meta-analysis had several limitations: inter-study heterogeneity of the probiotic supplement, the duration of treatment, the effective dose range, and a possible positive publication bias. Another limitation was that only fasting glucose was evaluated with a non-significant clinical minor effect, but not mean daily glucose or postprandial glucose levels, which better predict pregnancy complications in GDM (81). Nachum et al. in a multicenter placebo controlled RCT among women with GDM recently found no effect of a mixture of probiotic strains on maternal glycemic control, particularly preprandial and postprandial glucose values, and pregnancy outcomes (82).

## What is the fetal contribution to the development of gestational diabetes?

Maternal placental fetal interactions are the cornerstone for the development of GDM. The placenta is a fetal endocrine organ, which produces and secretes a variety of substances to adapt maternal physiology to support the growing fetus. The placenta contributes to maternal insulin resistance, ensuring that maternal blood glucose levels rise adequately to sustain fetal growth and a concurrent increase in maternal β-cell mass and function maintains maternal glucose hemostasis. A disruption in either process may result in GDM.

Placental proteins such as leptin and adiponectin, peptide hormones including human chorionic gonadotropin (hCG), human placental lactogen (hPL) and placental growth hormone (PGH) and steroid hormones progesterone and estrogen all significantly influence maternal glucose metabolism (83).

Placental endocrinology differs between women with and without GDM starting from the first trimester (84). It has been suggested that first trimester hCG or β-hCG concentrations negatively correlate with the risk for subsequent development of GDM. This finding was further (85) confirmed in *in vivo* models in which trophoblasts from pregnancies with GDM had decreased expression of genes associated with synthesis and differentiation of hCG (86).

PGH, a member of the somatotropin family, is unlike other placental hormones as it is secreted exclusively into the maternal circulation and in a continuous non-pulsatile fashion (87) and it is the only placental hormone directly affected by maternal glucose concentrations in a dose-dependent manner (88). PGH stimulates glucose-induced insulin secretion via the prolactin receptor and growth hormone receptor on maternal β-cells (89). Hyperglycemia leads to decreased PGH production, followed by a decline in insulin secretion, further worsening hyperglycemia in a self-propagating cycle. This process is especially prominent among women with GDM (90).

hPL is another member of the somatotropin family, detectable from week 6 of gestation and increasing progressively until the third trimester. Although hPL has been associated with increased maternal β-cell mass and function, acting via the prolactin receptor, and single nucleotide polymorphisms of the prolactin receptor gene PRLR have been associated with an increased risk of GDM (91), large studies have failed to demonstrate a difference in hPL levels between GDM pregnancies and controls (92, 93).

Leptin, whose main source of production in the non-pregnant state is the adipose tissue, increases steadily during pregnancy to peak at 28 weeks gestation due to a significant contribution from the placenta (94). Although leptin enhances peripheral insulin sensitivity and β-cell function and suppresses food intake via signaling satiety in the non-pregnant state (95), pregnancy is associated with progressive leptin insensitivity which may promote insulin insensitivity and glucose intolerance (94). Women with GDM have higher leptin levels than controls (92, 96, 97). Moreover, leptin may be secreted into fetal circulation, contributing to fetal growth and higher levels are observed in cord blood of macrosomic newborns compared to those with normal birthweight (96), an adverse outcome associated with GDM. Conversely, circulating adiponectin levels decline during pregnancy, with lowest levels in cases of rising BMI, insulin resistance and GDM (41) and several studies show that women with GDM have lower levels than controls (92, 96, 97).

Estrogens have demonstrated protective effects on β-cells, with estradiol (E2) promoting β-cell replication and neogenesis in animal models (98). Unconjugated estriol (E3), the most abundant estrogen in pregnancy, has been associated with GDM development when levels exceed the 95th centile in the early second trimester (99). Competitive binding of E3 and E2 to the estrogen receptor and decreased expression of both estrogen receptors in cases of GDM, which coincides with increased leptin expression and the associated increase in pro-inflammatory cytokines (100) may contribute to the development of insulin resistance.

Progesterone is associated with weight gain and fat deposition, but also with suppression of inflammatory responses (101). The precise effect of progesterone on β-cells remains elusive, but it has been suggested that insulin resistance results from reduced insulin binding, downregulation of the GLUT4 receptor (responsible for glucose uptake in muscle and adipose tissue) and insulin-induced hepatic gluconeogenesis (102). Conflicting studies have shown increased risk of GDM associated with lower (103) and higher levels of progesterone (104). Additionally, exogenous supplementation in the form of 17α-hydroxycaproate from prevention of preterm birth has also been demonstrated to increase the risk of GDM (105).

Fetal sex is also emerging as a contributor to the development of GDM with increased prevalence and severity noted in pregnancies with male fetuses (106). This risk has been estimated to be around 4% higher than pregnancies with female fetuses (107) and is also associated with an increased lifelong risk of type 2 DM (108). This may indicate that regulation of glucose metabolism may differ according to fetal sex. Some studies have noted higher insulin resistance (109) or higher fasting glucose, indicating decreased β-cell function (110), although some studies have observed no differences between pregnancies with male and female fetuses (111).

Pregnancies with a male fetus have lower average hCG levels throughout pregnancy (112) and lower maternal PGH levels (113), both associated with poorer control of glucose hemostasis. Additionally, male placentas are smaller despite their relative increased growth, and therefore assumed to be more efficient, although with less reserve to counteract metabolic challenges (114). Although no biologic models have been described to date, gene expression between male and female fetuses differs from early in pregnancy, and this, in combination with differing hormonal expression and function, should be studied for their contribution to the development of GDM.

## Prenatal ultrasound for the assessment of fetal effects of gestational diabetes

Ultrasound is a non-invasive test which facilitates the identification of various fetal complications associated with GDM. In this article, we will focus on the limitations of fetal weight assessment.

Sonographic estimation of fetal weight aids in the assessment of glucose control and for planning timing and mode of delivery. Some studies report no significant difference in the accuracy between clinical approximations and sonographic weight estimations, whereas others advocate that ultrasound estimation is superior (115–118). The increasing maternal BMI and alteration in the fetal biometrical measurements with growing thoraco-abdominal size in diabetic pregnancies may affect the accuracy of both methods.

Estimated fetal weight formulas are based on the measurements of head circumference, biparietal diameter, abdominal circumference, and femur length, either alone or in various combinations, of non-diabetic fetuses. In most studies there was no significant difference in the accuracy of fetal weight estimation in diabetic pregnancies as opposed to non-diabetic pregnancies. Recently several studies (119–121) have proposed that the Hadlock I formula (122) is superior in accuracy in cases of GDM, however, Cesnaite et al. (123) advocate the use of the Hsieh formula (124) and recommended to use a combination of formulas to improve the accuracy of estimation. Yet another approach suggests the use of abdominal circumference alone for macrosomic fetuses in GDM (125). There is a lack of consensus regarding timing and frequency of sonographic fetal weight measurements. One study found that a single estimation at 29 to 34 weeks gestation in poorly controlled GDM failed to identify mascrosomia at term (126), another has shown that serial measurements improve prediction (127).

Lee et al. (128, 129) suggested the inclusion of soft tissue development as part of a weight estimation procedure to improve the precision of fetal weight estimation. They added three-dimensional fractional thigh volume to two-dimensional sonographic measurements. Data suggest that the addition of three-dimensional measurements to conventional two- dimensional biometry was associated with improved weight predictions in diabetic pregnancies (130, 131). However, Tulli et al. found the Hadlock formula to be superior to the three-dimensional method for predicting birth weight and macrosomia in diabetic women (132).

Magnetic resonance imaging (MRI) has been reported as an alternative modality for fetal weight estimation (133, 134) and found to be accurate, but inaccessibility limits its’ routine use.

In summary, although ultrasound for estimated fetal weight in the third trimester of pregnancy is a common practice, its ability to diagnose macrosomia accurately in diabetic pregnancies is insufficient. Therefore, caregivers should be alert to all risk factors and to the inherent inaccuracy of fetal weight estimation to determine timing and route of delivery.

## Screening

Screening for GDM is a standard part of antenatal care and is based on a 1-hour 50g glucose challenge test (GCT). In their original study, O’Sullivan et al. (135) found a cut-off of 130mg/dL to be moderately sensitive and specific for GDM, and more importantly to have a high negative predictive value of 99.4%. Following these findings, several studies have evaluated cut-offs between 130 and 140mg/dL with no consensus regarding the optimal threshold to improve pregnancy outcomes (136). Increasing the threshold of maternal glucose concentration for diagnostic testing to 140mg/dL yielded higher positive predicting values and lower false positive rates, unnecessary additional testing and its’ associated cost and patient anxiety across various racial and ethnic groups (137). This, however, decreases sensitivity, although this effect may be negligible (137).

The original recommendations from the American College of Obstetricians and Gynecologists (ACOG) in 1986 proposed screening only for women with risk factors defined as age >25 years, high risk ethnicity or first-degree relative with DM (138). However, these criteria resulted in a failure to detect up to 50% of cases. Subsequently since 2014 the US Preventative Task Force recommends screening for GDM to all pregnant women at or beyond 24 weeks gestation (139).

**Figure 3:**
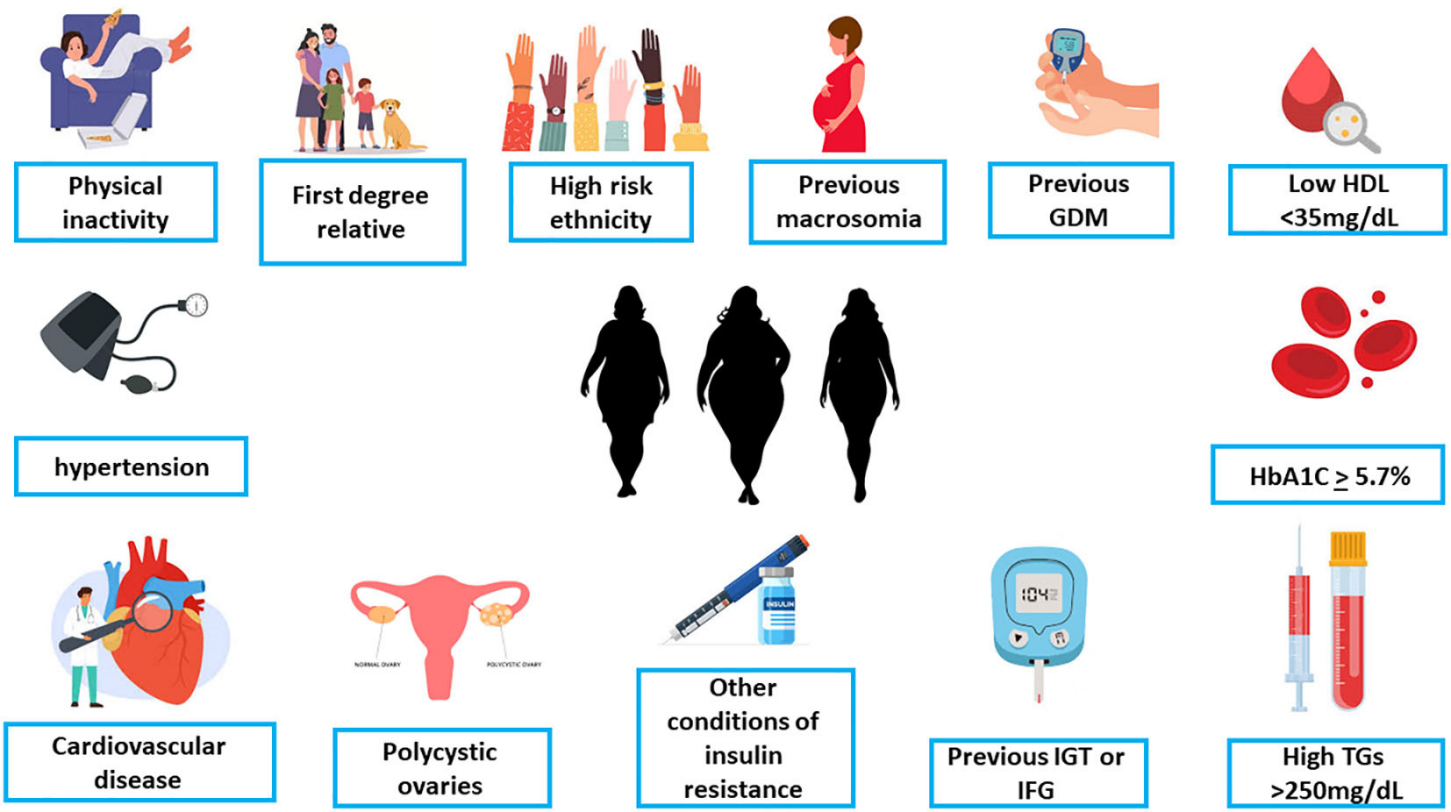
The criteria for early screening for gestational diabetes in overweight/obese women with at least one additional risk factor (ADA, [142 with permission]). GDM, Gestational diabetes mellitus; HDL, high-density lipoproteins; HbA1C, hemoglobin A1C; TG, triglycerides; IGT, impaired glucose tolerance; IFG, impaired fasting glucose.

As a proportion of women presenting with GDM will in fact turn out to be undiagnosed pre-gestational DM, the American Diabetes Association (ADA) suggests early screening with diagnosis of pregnancy for overweight or obese women with additional risk factors (140), (**Figure 3**), followed by additional testing at 24-28 weeks gestation for those who screened negative in the beginning of pregnancy (141). However, early-onset GDM diagnosed before 24 weeks was not associated with elevated rate of fetal malformations, but with higher risks for macrosomia, shoulder dystocia, and neonatal hypoglycemia (142). These women should undergo strict glycemic control, intensive monitoring, and careful neonatal evaluation.

### Early screening

Early screening in pregnancy should be considered if the patient is overweight (BMI ≥ 25) and has at least one additional risk factor such as - history of GDM or macrosomia, family history of diabetes, HbA1c ≥ 5.7%, impaired glucose tolerance impaired fasting glucose, conditions associated with metabolic syndrome (hypertension, low HDL cholesterol levels, elevated triglyceride), conditions associated with insulin resistance including polycystic ovary syndrome and morbid obesity, age of 35 and above, cardiovascular disease, 1st degree family history of diabetes and other factors that may exhibit as risk factor for pregestational diabetes (140, 143).

Noteworthy, early pregnancy evaluation may reveal women with pregestational diabetes. In accordance with the American Diabetes Association the diagnosis of pregestational diabetes is confirmed if one of the following thresholds is met: fasting plasma glucose ≥ 126 mg/dL (7.0 mmol/L), 2-hour plasma glucose ≥ 200 mg/dL (11.1 mmol/L) during a 75-gram oral glucose tolerance test, HbA1c ≥ 6.5% or random plasma glucose ≥ 2—mg/dL (11.1 mmol/L) in the presence of classic hyperglycemic symptoms (140). Yefet et al in a population-based retrospective cohort study, evaluated 219 women with GDM diagnosed prior to the 24th week. Type 2 diabetes was diagnosed in 11 (5%) women post pregnancy. The most accurate and the only independent marker for undiagnosed type 2 diabetes was HbA1c ≥ 5.8% (sensitivity of 89%, specificity of 86%, 99.4% negative predictive value, and 23% positive predictive value) (144). They concluded that early-diagnosed GDM with HbA1c of 5.8% or more should be managed as type 2 diabetes mellitus, and after delivery the definitive diagnosis should be made.

### Diagnosis

Diagnosis of GDM is made using the 100g 3-hour oral glucose tolerance test (OGTT), performed in women who screened positive. It is important to note that in some cases GDM can be diagnosed following a screening GCT if plasma glucose levels exceed 200mg/dL. The initial diagnostic criteria for GDM (135) were established in 1964 based on two or more pathological venous blood glucose values (**Table 2**), which correlated with elevated risk of developing type 2 DM postpartum, and later were found to be associated with fetal and neonatal complications. Following this study, the National Diabetes Data Group (NDDG) suggested criteria for plasma glucose concentrations (145) and in 1994 the ACOG adopted the criteria of Carpenter and Coustan (138, 146) all of which are presented in **Table 2**. Treatment of GDM diagnosed by the later criteria led to improved pregnancy outcomes (147).

**Table 2:** Diagnostic criteria for oral glucose tolerance test in the USA.

Time	O’Sullivan and Mahan (1964) 100g OGTT (venous blood)	NDDG (1979) 100g OGTT (plasma glucose concentrations)	Carpenter and Coustan (1982) 100g OGTT (plasma glucose concentrations)	IADPSG (2010) 75g OGTT (plasma glucose concentrations)
0’ (fasting)	90	105	95	92
60’	165	190	180	180
120’	145	165	155	153
180’	125	145	140	————————

OGTT, oral glucose tolerance test; NDDG, The National Diabetes Data Group; IADPSG, The International Association of Diabetes and Pregnancy Study Groups.

The HAPO study (148) in 2008 attempted to establish new GDM criteria centering around the risk of adverse pregnancy outcomes. Based on a one-step diagnostic 2-hour 75g OGTT with no prior screening, correlation between glucose concentrations and GDM-related complications was identified. The criteria for GDM diagnosis were set by the International Association of the Diabetes and Pregnancy Study Groups (IADPSG) (**Table 2**). The implementation of the IADPSG GDM diagnostic criteria led to a significant increase in the number of women diagnosed as GDM without improving pregnancy outcomes (149–152). These reports have led the National Institute of Health (NIH) to conclude that there was insufficient evidence to adopt the one-step diagnostic approach to GDM, citing particular concern regarding the increase in the prevalence of GDM, its’ costs and interventions (136, 153, 154).

More recently it has been shown that women with just one abnormal value on the 3-hour 100g OGTT also have elevated risk for adverse perinatal outcomes (155, 156), and a randomized trial has shown that maintaining tight glycemic control with diet and insulin may be beneficial in preventing GDM-associated complications (157). Following this, the ACOG has recently approved the use of one abnormal value for the diagnosis of GDM (158).

The current recommendations for the diagnosis of GDM varies among professional societies in the USA (**Table 2**) and globally (**Supplementary Table 1**).

### Glucose monitoring

No controlled trials have been performed to identify optimal glycemic targets. The ADA and ACOG recommend that fasting or pre-prandial blood glucose values be below 95 mg/dL and post prandial blood glucose values be below 140 mg/dL at 1 hour or 120 mg/dL at 2 hours to reduce the risk of macrosomia (143, 158). No study to date has demonstrated the superiority of either approach (159–162), and this may be because postprandial glucose peaks at approximately 90 minutes, between the two times points using 130 mg/dL as a cutoff of two standard deviations above the mean (163).

Nachum et al. found that ambulatory care was more effective than hospitalization among GDM patients regarding glycemic control and neonatal morbidity (164). This was not only more convenient for the pregnant diabetic patient, but significantly reduced treatment costs.

### Treatment

Several trials had proven the efficiency and importance of treatment and glucose control on pregnancy outcomes. In a randomized control study published in 2005, treatment significantly reduced the rate of serious perinatal outcomes (defined as death, shoulder dystocia, bone fracture and nerve palsy) from 4 percent to 1 percent (165). Later, Landon et al. (147), showed that well controlled glucose lowered the risks of fetal overgrowth, shoulder dystocia, cesarean delivery, and preeclampsia. In a recent RCT, early versus late treatment of GDM before and after 20 weeks’ gestation led to only a modestly lower incidence of a composite of adverse neonatal outcomes. Furthermore, no material differences were observed for neonatal lean body mass or pregnancy-related hypertension (166).

Intensified versus conventional management of GDM using insulin treatment evaluated by Langer et al. (167) and Nachum et al. (168) resulted in better glucose control and enhanced perinatal outcome.

When diet and lifestyle change such as exercise fail to control the glucose (169), pharmacological treatment with insulin or oral metformin or glyburide is needed.

Historically, insulin was the main pharmacological treatment for GDM. In 2000 Langer et al. compared in a RCT insulin to glyburide, a second-generation sulfonylurea that binds to pancreatic beta-cell adenosine triphosphate potassium channel receptors to increase insulin secretion and insulin sensitivity of peripheral tissues (170). They found that glyburide was a clinically effective alternative to insulin therapy. They also reported that glyburide was not detected in the cord serum of any infant in the glyburide group. Other former studies revealed as well that glyburide does not cross the placenta (171, 172).

Since then, a large nationwide retrospective cohort study in the USA, including 10,778 women pharmacologically treated for GDM, the use of glyburide increased from 7% in 2000 to 65% in 2011, and has become a widespread treatment since 2007 (170).

Several guidelines considered oral agents as an acceptable first-line treatment in select patients, such as those with normal fasting blood glucose levels and modest postprandial hyperglycemia (171, 172).

Metformin, a biguanide that stimulates glucose uptake in peripheral tissues, inhibits hepatic gluconeogenesis and glucose absorption. It has been shown to freely cross the placental barrier (173). Several studies have reported outcomes in women exposed to metformin during pregnancy and did not reveal any adverse outcomes including congenital malformations and neonatal hypoglycemia (174–177). A large key RCT compared in 2008 metformin to insulin for the treatment of 751 women with GDM (178). Both groups experienced similar rates of a composite outcome of perinatal morbidity, consisting of neonatal hypoglycemia, need for phototherapy, birth trauma, respiratory distress, prematurity, and low Apgar scores. In the metformin group, 46% received supplemental insulin. In another prospective trial, women randomized to metformin had lower mean glucose levels, less gestational weight gain, and neonates with lower rates of hypoglycemia than those randomized to insulin (179). Another recent RCT found that metformin compared with insulin treatment was associated with a better postprandial glycemic control for some meals, a lower risk of hypoglycemic episodes, less maternal weight gain, and a low rate of failure as an isolated treatment. Most obstetrical and perinatal outcomes were similar between groups (180).

Large meta-analysis published in 2015, which compared glibenclamide (glyburide), metformin, and insulin for the treatment of GDM, analyzed 15 articles including 2509 subjects (181). Glibenclamide was found to be inferior to both insulin and metformin, regarding maternal weight gain, LGA, macrosomia and neonatal hypoglycemia. Metformin (plus insulin when required) performed slightly better than insulin. The authors concluded that glibenclamide should not be used for the treatment of women with GDM if insulin or metformin is available. Another large meta-analysis of 4982 women with GDM who were treated with glyburide and 4191 treated with insulin, newborns of women treated with glyburide were at increased risk for neonatal intensive care unit (NICU) admission (risk ratio, RR=1.4), respiratory distress (RR=1.6), hypoglycemia (RR=1.4), birth injury (RR=1.4) and LGA (RR=1.4), compared with those treated with insulin (182). A recent large RCT (N=914) found glyburide inferior to insulin in preventing perinatal complications (183). They concluded that glyburide should not be used as a first-line treatment.

Meta-analyses comparing the use of oral agents with insulin therapy have found that both strategies were acceptable and may improve pregnancy outcomes in patients with GDM (176, 181, 184–189). Nevertheless, the most recent meta-analysis revealed that maternal randomization to glyburide resulted in heavier neonates with a propensity to increased adiposity versus insulin- or metformin-exposed groups. Metformin-exposed neonates were lighter with reduced lean mass versus insulin- or glyburide-exposed groups, independent of maternal glycemic control (187). Additional meta-analysis, which examined short and long-term outcomes of metformin compared with insulin, demonstrated a reduced risk for neonatal hypoglycemia and pregnancy-induced hypertension (188).

Nachum et al. compared the efficacy and safety of glyburide versus metformin and their combination for the treatment of GDM (190). In the glyburide group, 17% of patients were eventually treated with insulin compared with only 4% in the metformin group (P = 0.03). The combination of the drugs significantly reduced the need for insulin from 32% to 11% of patients. Mean daily blood glucose and other obstetrical and neonatal outcomes were comparable between groups.

Both metformin and glyburide have a similar treatment failure rate and the same need for supplemental treatment such as insulin, ranging from 15% to 30% (189, 190).

Whether oral antidiabetic medications during pregnancy may affect the progression to type 2 diabetes later in life, is not yet known. A Cochrane meta-analysis, reporting data on more than 7000 women, which compared insulin to oral antidiabetic pharmacological therapies (mainly metformin and glyburide), found similar effects on health outcomes (186).

The long-term metabolic influence of metformin on the offspring is crucial (173). A follow-up study (178) found similar developmental outcomes by 2 years of age (191). A recent meta-analysis revealed reasuring results: in children up to the age of 14 years, metformin usage during pregnancy was not associated with adverse neurodevelopmental outcomes (192). A possible explanation for the unfavorable outcomes with glyburide treatment is that in contradiction to earlier studies reporting that it does not cross the placenta considerably (170, 193), more recent studies that used more sensitive methods to detect plasma concentrations of glyburide found that glyburide readily crosses the placenta, reaching 50–70% of total maternal plasma concentration with similar average concentrations of maternal and umbilical cord plasma of the unbound fraction and greater than the maternal plasma concentration in 20–37% of the samples (194, 195). Thus, glyburide might lead to fetal overgrowth and neonatal hypoglycemia through excessive fetal insulin secretion, whereas metformin increases insulin sensitivity in the target organs without causing hyperinsulinism.

In a recent study, between 2015 and 2018 in the USA, insulin (26 to 44%) followed by metformin (17 to 29%) has replaced glyburide (58 to 27%) as the most common pharmacotherapy for GDM among a privately insured US population during a time of evolving professional guidelines (196).

The ADA and ACOG still consider insulin the preferred treatment when pharmacologic treatment of GDM is indicated (143, 158). Nevertheless, the ACOG recognizes that in women who decline insulin therapy or who the obstetricians believe will be unable to safely administer insulin, or for women who cannot afford insulin, metformin (and rarely glyburide) is a reasonable alternative (143). Moreover, the Society of Maternal-Fetal Medicine stated that metformin may be used as a first line treatment for GDM (197).

### Recurrence

The reported recurrence rate of GDM had a wide range from 30% to 84% (198–205). Schwartz et al. conducted a meta-analysis of 18 studies with 19,053 participants to evaluate the rate and risk factors of recurrence (206). The overall recurrence rate of GDM was forty eight percent (CI 95%, 41 – 54%) (206).

Primiparous women had a lower recurrence rate compared with multiparous women (40% and 73%, respectively). A significant association between ethnicity and GDM recurrence rate was found. Non-Hispanic whites had lower recurrence rate compared with other ethnicities (39% and 56%, respectively). No evidence for an association between family history of diabetes and GDM recurrence was found. A follow-up meta-analysis revealed that women with GDM recurrence were older, heavier, had higher 100-g OGTT levels and higher weight gain between pregnancies. Insulin use and fetal macrosomia were also associated with GDM recurrence (207). Weight gain between pregnancies and inter-pregnancy interval were found to be modifiable risk factors for GDM recurrence. A model that was developed predicted that reducing the inter-pregnancy interval and weight gain between pregnancies can reduce substantially the risk of GDM recurrence (208). Another important modifiable factor for recurrence may be glucose control during pregnancy. A retrospective population-based cohort study of 426 women with first diagnosed GDM pregnancy was conducted. The analyses revealed that the 2-hourpostprandial levels among women with GDM recurrence were substantially higher throughout gestation (209). This may imply that tighter postprandial glycemic control may prevent GDM recurrence. GDM recurrence was associated with increased risk for type 2 DM (210).

### Delivery

Optimally timing of delivery should be postponed until the fetus is fully matured, given that antepartum fetal surveillance is reassuring and GDM is well controlled (211). Decision of labor induction should incorporate trade-offs between the increased risk of neonatal morbidity in early term neonates, between 37 + 0 weeks and 38 + 6 weeks and between the ongoing risk of still birth and delivery related complication of fetal overgrowth as shoulder dystocia and caesarean delivery (211). Delivering women with GDM at 38 weeks or 39 weeks of gestation would reduce overall perinatal mortality without increasing cesarean delivery rate (158).

#### Well controlled GDM A1 (diet treated)

Expectant management is appropriate up to 40 6/7 weeks of gestation, unless otherwise is indicated for another problem such as concurrent hypertensive disorders (158). Labor induction should be offered by 39 + 0 weeks, while discussing the risks and benefits of induction versus expectant management (158).

#### GDM A2 (pharmacologically treated)

##### Well controlled

delivery is recommended from 39 0/7 weeks to 39 6/7 weeks of gestation (158).

##### Poorly controlled

the literature lacks clear guidance about the degree of glycemic control that necessitates early induction, but delivery between 37 0/7 weeks and 38 6/7 may be conducted. In case of failure in-hospital attempts of glycemic control or if an abnormal fetal testing is present, delivery in the late preterm period can be considered (158).

#### Cesarean delivery

Cesarean delivery should be suggested when the estimated fetal weight is 4000 – 4500 grams considering the obstetrical history and clinical assessment of the female pelvis (211). Furthermore, the ACOG recommends caesarean delivery when estimated fetal weight exceeds 4500gr (158). During labor, in case of arrest of dilatation or descent despite adequate contractions, the possibility of cephalopelvic disproportion should be raised.

Shoulder dystocia is encountered in roughly 25% of delivery of macrosomic infants complicated by a prolonged second stage of labor. Hence, in case of protracted labor or failure of descent, caesarean delivery should be considered (211).

### Short and long-term complication of the mother and offspring

#### Maternal short-term complications

Women with GDM have a higher risk of developing preeclampsia and undergoing a cesarean delivery (158). GDM was associated with infections (pooled-OR 1.3 95% CI [1.2-1.5]), specially with SARS-CoV-2 (pooled-OR 1.5 95% CI [1.2-2.0]), bacterial infections (pooled-OR were 1.2 95% CI [1.1-1.4]), and urinary tract infections (pooled-OR of 1.2 95% CI [1.1-1.3]), but not with vaginal candidiasis or gingivitis (212).

#### Maternal long-term complications

##### Long-term complications

It is known that risks associated with GDM extend beyond pregnancy and the neonatal period. The history of GDM is a strong predictor of an increased risk for developing type 2 DM, including diabetes-related vascular disease, as well as components of metabolic syndrome (213, 214).

##### Impaired glucose tolerance

During the early postpartum period, approximately 30% of patients with GDM have impaired glucose tolerance (215).

##### Type 2 DM

Many studies estimated that 50% of patients suffering from GDM will eventually develop type 2 DM (216). Women with GDM had a 10-fold higher risk of developing type 2 DM during a 10-years follow up period as compared to women with no GDM (217). A revealed that review of 28 studies has demonstrated a rapid increase in the incidence of type 2 DM especially in the first 5 years after delivery (17% to 50%) (218). Greenberg et al. (219), reported an increased risk for type 2 DM in women with any 2-h postprandial blood glucose level of 150 mg/dl or higher during pregnancy.

Within six months at postpartum, studies demonstrated defects in insulin secretory response and decreased insulin sensitivity in women with GDM (220). This emphasizes the importance of increased interest on the profound risk of developing type 2 DM after GDM, also warrants initiatives shortly after delivery including lifestyle, dietary and pharmacological interventions aimed to prevent or delay the development of type 2 DM (220).

The risk of future maternal Type 2 DM is increased with the number of abnormal OGTT values and was highest among women having three abnormal values yielding an incidence rate of 50 per 1000 persons per year. The type of OGTT abnormality affects the calculated risk, as women with abnormal fasting glucose had the utmost risk, while an abnormal 2-hours value was associated with the lowermost risk (221).

**Figure 4:**
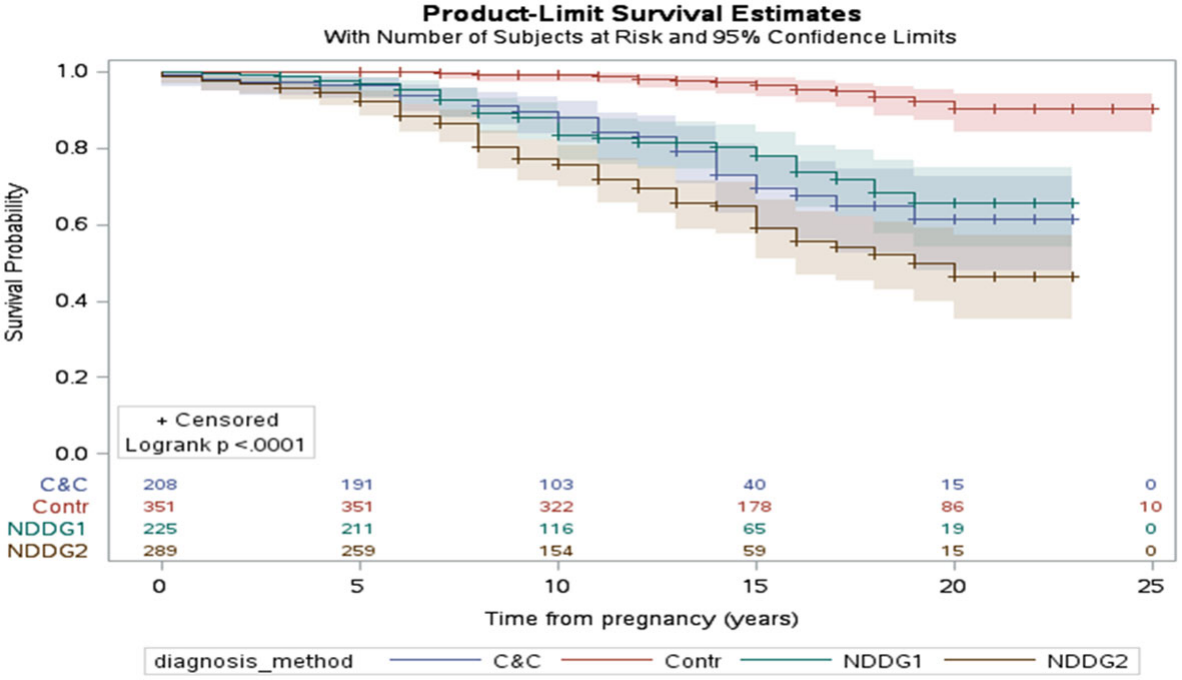
The interval in years between pregnancy with GDM and the development of type 2 diabetes mellitus (Kaplan–Meier survival curve). The impact of the diagnostic method for GDM is highlighted in the figure. The control group (Contr) were women without gestational diabetes. NDDG1 and NDDG2 are women with one and two abnormal values according to the National Diabetes Data Group criteria, respectively. C&C Carpenter and Coustan, GDM gestational diabetes mellitus, NDDG National Diabetes Data Group [222 with permission].

Furthermore, diagnosis by two abnormal values of the NDDG criteria carried the highest risk of 31%, compared with 19% with one abnormal value of the NDDG criteria, 18% by Carpenter and Coustan criteria and 5% in the controls during a mean follow-up of 12.4 ± 5.3 years (222, **Figure 4**). Noteworthy, Parity, fasting glucose, 1 hour value of the OGTT and insulin use were all independent risk factors (222).

##### Metabolic syndrome

GDM can be a risk factor for developing metabolic syndrome including central obesity, hypertriglyceridemia, low HDL levels, hyperglycemia, and hypertension. The prevalence of the metabolic syndrome is 2–4 times higher in women with prior GDM and more likely to develop at the ≥3 months postpartum (212).

Additional prospective observational follow-up study of patients with mild GDM (normal fasting glucose level on OGTT), revealed that metabolic syndrome was common, being diagnosed in 33% of these women at 5–10 years (223).

Antenatal glucose measurements and BMI should be used to select women with a higher risk of developing metabolic syndrome, so the condition with its subsequent cardiovascular outcomes can be sought out more effectively and prevented through lifestyle modifications, weight loss and medical intervention when needed (224, 225).

##### Cardiovascular disease

Patients with GDM are more prone to develop CVD that includes myocardial infarction and stroke, even at a younger age than those with no history of GDM (226). Changes in hemodynamics among GDM women, such as increased arterial stiffness and blood pressure, was demonstrated in these women (227). A pooled analysis of nine studies including over five million females and more than 101,000 cardiovascular events, those with GDM had a two-fold higher risk of future CVD compared with those with no history of GDM (228). This risk was not dependent upon the development of type 2 DM (228). The risk of CVD in women with GDM is evident by as early as the first decade after delivery.

**Figure 5:**
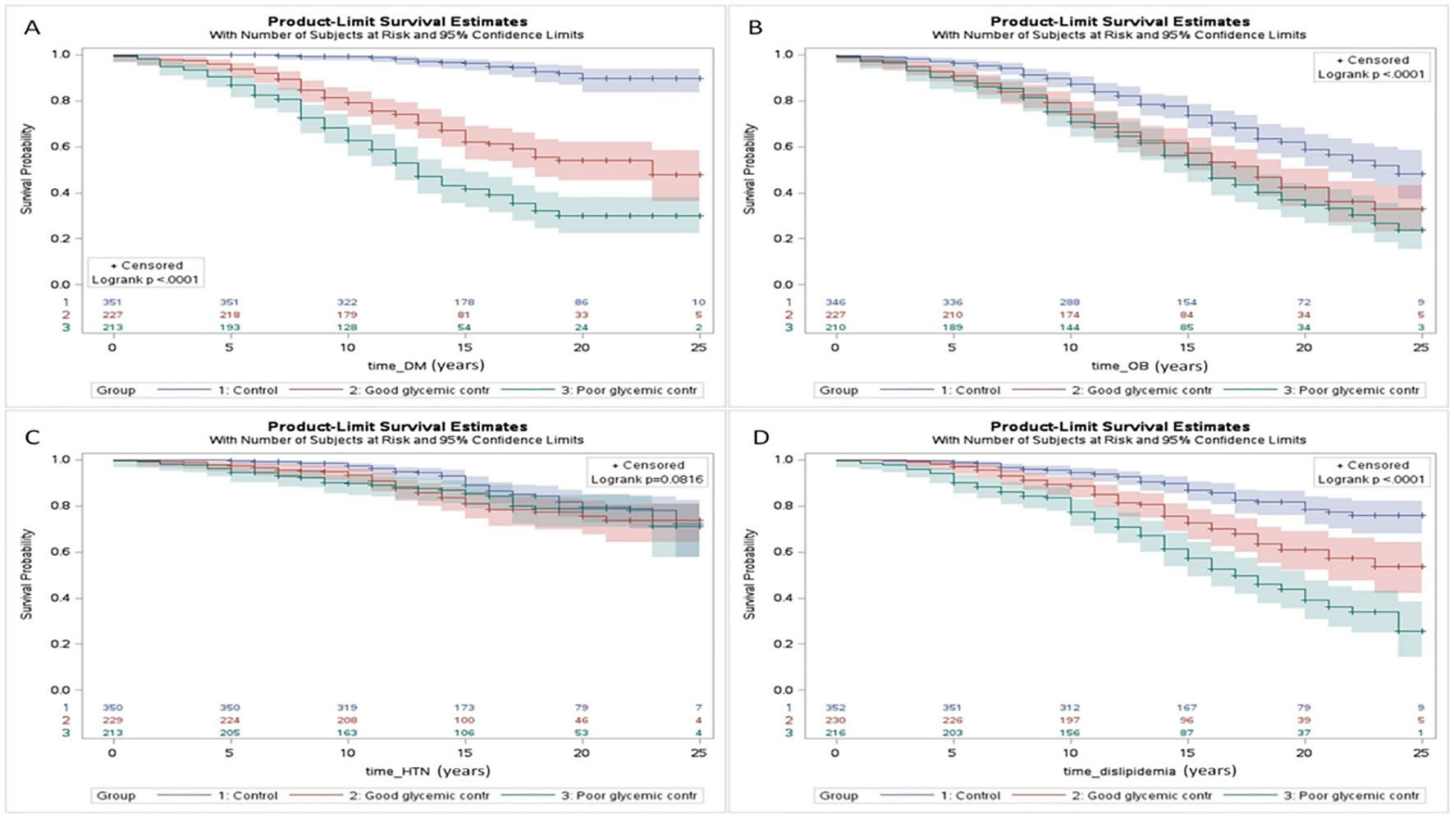
Survival analysis (Kaplan Meier curves) of the interval (years) from pregnancy until the development of chronic maternal morbidity including: type 2 diabetes mellitus (DM) **(A)**, obesity (OB) **(B)**, chronic hypertension (HTN) **(C)**, and dyslipidemia **(D)** for women in the: 1) control group; 2) GDM with good glycemic control; and 3) GDM with poor glycemic control. A statistical significance was found for all the comparisons (p < 0.05) further emphasizing the impact of gestational; diabetes on future maternal health and cardiometabolic morbidity. Control refers to women without gestational diabetes mellitus. Excluded from analysis women with the diagnosis prior to the index pregnancy or cases in which the exact date of the diagnosis is unknown [225 with permission].

Yefet et al. (225) demonstrated that glycemic control in GDM is an important independent risk factor for future type 2 diabetes mellitus and dyslipidemia as a part of the metabolic syndrome and CVD (**Figure 5**). The fact that it is still statistically significant after controlling for the OGTT results, which reflect the baseline disease severity, implies that improving glycemic control might reduce the risk for those outcomes.

#### Fetal and neonatal short-term complications

##### Macrosomia

Complicates as many as 50% of pregnancies in women with GDM. Delivery of an infant weighing greater than 4500 g occurs 10 times more often in women with GDM compared with a population of women with normal glucose tolerance (158). Deliveries of LGA infants are at increased risk for shoulder dystocia, birth trauma and Cesarean section. Insulin is the most important fetal growth hormone, and fetal hyperinsulinemia results in excessive fetal growth. Infants of mothers with GDM have an increase in fat mass, in addition, their growth is disproportionate. Both GDM and obesity are associated with an increased risk of LGA. In one report, the prevalence of LGA among women with GDM who had normal weight versus those who were obese was 14% and 22%, respectively (229). The results of several clinical trials have shown that tight maternal glycemic control has been associated with a reduction in macrosomia and in fat mass (158). Landon et al. (147) reported a rate of 9% for macrosomia when mean glucose values were less than 110 mg/dL compared with 34% when less optimal control was achieved (147).

##### Hypoglycemia

Neonatal hypoglycemia is defined as a blood glucose level less than 35 to 40 mg/dL during the first 12 hours of life. Hypoglycemia is a by product of hyperinsulinemia and is common in macrosomic newborns. The hyperinsulinemic state usually lasts for 2-4 days. A strict monitoring of blood glucose level is obligatory for the hypoglycemic infants, as some may need glucose supplementation.

Infants of mothers with diabetes who are preterm or small for gestational age (SGA) are at more increased risk for persistent hypoglycemia. Strict glycemic control during pregnancy may help to reduce the risk of neonatal hypoglycemia (229).

##### Stillbirth

Patients with GDM and suboptimal glucose control appear to have an increased risk of stillbirth compared with the general obstetric population. Excessive stillbirth rates in pregnancies complicated by diabetes have been linked to chronic intrauterine hypoxia (158).

Infants of mothers with diabetes are at increased risk for intrauterine or perinatal asphyxia due to macrosomia and its consequences, as well as cardiomyopathy. This can result in low Apgar scores and intrauterine fetal death. A previous study of 162 infants of mothers with diabetes, 27% had perinatal asphyxia (230).

##### Neonatal metabolic disorders

Neonates of pregnancies complicated by GDM have been reported to be at increased risk of multiple, often transient disorders including hyperbilirubinemia, hypocalcemia, hypomagnesemia, and polycythemia.

##### Hypocalcemia

Defined as a total serum calcium concentration less than 7 mg/dL or an ionized calcium value less than 4 mg/dL. The prevalence of hypocalcemia ranges from at least 5% and up to 30% in infants of mothers with diabetes. The lowest serum calcium concentration typically occurs between 24 - 72 h after birth. Hypocalcemia usually is asymptomatic and resolves without treatment. On the other hand, when it is symptomatic, it can cause jitteriness, lethargy, apnea, tachypnea, or seizures (158). Good glycemic control during pregnancy is associated with a reduction in the rate of neonatal hypocalcemia (231).

##### Hypomagnesemia

Defined as serum magnesium concentration less than 1.5 mg. It can occur in up to 40% of infants of mothers with diabetes within the first three days after birth. It is usually transient and asymptomatic and, therefore it is not treated (232).

##### Hyperbilirubinemia

Hyperbilirubinemia is frequently observed in 38% of pregnancies in women with GDM, especially in preterm infants. In addition to prematurity, other factors associated with neonatal jaundice include poor maternal glycemic control, macrosomia, and polycythemia (158).

##### Polycythemia and hyperviscosity syndrome

Hematocrit of more than 65% are more likely in infants of mothers with diabetes. The underlying pathway of polycythemia and hyperbilirubinemia most likely involves increased red blood cells production, which is stimulated by increased erythropoietin production caused by chronic fetal hypoxemia and fetal exposure to oxidative stress (233). Polycythemia may lead to hyperviscosity syndrome. Untreated neonatal polycythemia may promote vascular sludging, ischemia, and infarction of vital tissues, such as renal vein thrombosis seen in these infants (225).

##### Cardiomyopathy

Infants of mothers with diabetes are at increased risk for transient hypertrophic cardiomyopathy. The most seen change is thickening of the interventricular septum, resulting in potential obstruction of left ventricular outflow. Fetal hyperinsulinemia, which increases the synthesis and deposition of fat and glycogen in the myocardial cells, is thought to be the cause of cardiac hypertrophy. It is most likely to occur in mothers with poor glycemic control during pregnancy. Infants often are asymptomatic, but 5–10% of them have respiratory distress, signs of poor cardiac output or heart failure (230). Symptomatic infants typically recover after 2-3 weeks of supportive care, and echocardiographic findings resolve within 6 -12 months (234). The risk of cardiovascular hospitalizations of the offspring is 0.97% for GDM A2 versus 0.57% for GDM A1 as compared with 0.33% for controls with no GDM, respectively; p < 0.001 (235).

##### Respiratory distress and NICU admission

At animal studies, there were evidence that hyperglycemia and hyperinsulinemia can affect pulmonary surfactant biosynthesis. Cortisol induces synthesis of fibroblast-pneumocyte factor on pulmonary fibroblasts (230). Clinical studies produced conflicting data. A secondary analysis of the Antenatal Late Preterm Delivery Steroids trial reported that neonates born to women with GDM were no more likely to meet the primary outcome (neonatal respiratory morbidity in the first 72 hours of life) than those born to women without GDM, even after adjusting for confounding variables in age, parity, and hypertensive disorders of pregnancy (12.1% vs 13.1%, adjusted RR 0.84; 95% CI 0.61-1.17), nor were they more likely to have severe respiratory complications or prolonged NICU admissions (236). This report included only late preterm births (gestational age of 34 0/7 - 36 5/7 weeks) and did not provide information about each participant’s glucose control and diabetes treatment. Good glycemic control could have lowered the risk of respiratory problems in the infants. In addition to RDS, other causes of respiratory distress in infants of mothers with GDM may include transient tachypnea of the newborn and cardiomyopathy (236).

#### Long-term neonatal outcomes

Prenatal exposure to high levels of glucose made the risk of postnatal metabolic complications higher, and even impacted neurodevelopmental outcome.

##### Diabetes and glucose intolerance

Intrauterine exposure to maternal diabetes is strongly associated with risk of developing type 2 DM, overall, 47% (95% CI 31-64) of type 2 diabetes in youth could be attributed to intrauterine exposure to maternal diabetes and obesity, compared to 6% of nondiabetic youth controls (237). Offspring of mothers with GDM history have at least 5 times greater risk of developing impaired glucose tolerance than those not exposed to gestational diabetes (235). One study concluded that obese youth exposed *in utero* to GDM show early inability of the beta cell to compensate adequately in response to decreasing levels of insulin sensitivity. Of the youth that were not exposed to GDM 9% developed either impaired glucose intolerance or type 2 diabetes compared with 31% of the youth that were exposed to GDM. Exposure to GDM was the most significant predictor of developing impaired glucose intolerance or type 2 diabetes (238).

##### Obesity

A strong relationship was found between prenatal exposure to maternal diabetes and increased childhood BMI. Intrauterine exposure to hyperglycemia resulting in fetal hyperinsulinemia may affect the development of adipose tissue and pancreatic beta cells. One study found that children exposed *in utero* to GDM had a higher rate of long- term hospitalizations with diagnosis of endocrine morbidity (such as DM and obesity) compared to those unexposed. The rate of obesity following *in-utero* exposure to GDM was approximately 4.9% following GDM A1 and 7.8% following GDM uncontrolled by diet. The rates of obesity in children of non-GDM women was only 1.8% (P<0.001) (239).

##### Cardiometabolic sequelae

GDM is associated with 19% increased risk for the offspring to develop early onset cardiovascular disease. It is suggested that GDM is correlated with both functional and structural alteration of the fetal heart (240) that along with abnormal metabolic imprinting associated with diabetes leads to increased risk for early development of cardiovascular morbidity during adult life. This is part of the transgenerational effect of gestational diabetes, affecting not only the mother but also the offspring.

##### Neurodevelopmental outcomes

Little is known regarding the effect of maternal GDM on the subsequent long-term neurodevelopmental outcome of the offspring. A review reported that maternal GDM seems to have a negative impact on offspring’s childhood cognitive development, particularly language development (241). A population-based cohort study was conducted to assess whether *in utero* exposure to GDM increases the risk of long-term neuropsychiatric morbidity in the offspring. During the study period 231,271 deliveries met the inclusion criteria; 5.4% of the births were to mothers diagnosed with GDM (n = 12,642), of these 4.3% had GDM A1 (n = 10,076) and 1.1% had GDM A2 (n = 2566). A significant linear association was found between the severity of the GDM (no GDM, GDMA1, GDMA2) and subsequent neuropsychiatric disease of the offspring (1.02%, 1.36%, 1.68%, P<0.001, respectively) (242).

Findings also pointed to a possible association between *in utero* exposure to GDM and autistic spectrum disorder of the offspring (242).

## Summary and future frontiers

GDM is now the leading medical complication during pregnancy having significant short and long-term implications for the mother and offspring. Although extensive research has been done, substantial gaps still exist.

The usage of several probiotics did not improve glucose control of GDM, but other probiotics may be found in the future to be beneficial, and the role of the microbiome and other factors in the pathophysiology of GDM needs further clarification.

The two-step approach for screening and diagnosis was found to be superior to the one step approach. For screening, a GCT cutoff of 130-140 mg/dL is used, and for diagnosis a 100 grams 3-hour OGTT using at least two abnormal values of Carpenter and Coustan or one abnormal value of the NDDG criteria. The preferred GCT cutoff between 130 and 140 mg/dL and the significance of one abnormal Carpenter and Coustan criteria or different cutoffs for the 75 grams 2-hour OGTT criteria (such as odd ratio of 2 for LGA) are yet to be studied.

Fasting and postprandial glucose should be measured, but should other pre-prandial values be taken, how frequent and when to test the postprandial value (60, 90 or 120 minutes)? The level of glucose control seems to modify short and long-term complications for the mother and child, but what is the optimal glucose profile to prevent those complications is not known yet.

Glyburide was found to be less effective in preventing GDM complications in comparison with insulin and metformin, probably because it readily crosses the placenta, and should not be used as the first-line treatment. Is it safe enough for metformin to become the first-line treatment with the adjunction of insulin or glyburide as a second line?

How to improve our ability to estimate the fetal weight to optimize GDM management, and time and mode of delivery? When should we induce labor before 39 weeks and what is the preferred estimated fetal weight (or other criteria) to recommend cesarean section?

We have come a long way, but these challenges are still there and much more research work should be done to answer those important questions for the patients, the clinicians, and the researchers.

## References

[1] Battat TL Erez O. Spontaneous preterm birth: a fetal-maternal metabolic imbalance. Maternal-Fetal Med. (2023) 5:223–8. doi: 10.1097/FM9.0000000000000205 PMC12094355 40406555

[2] Romero R. Prenatal medicine: the child is the father of the man. 1996. J. Matern Fetal Neonatal Med. (2009) 22:636–9. doi: 10.1080/14767050902784171 19736614

[3] Carpenter MW Canick JA Hogan JW Shellum C Somers M Star JA. Amniotic fluid insulin at 14-20 weeks’ gestation: association with later maternal glucose intolerance and birth macrosomia. Diabetes Care. (2001) 24:1259–63. doi: 10.2337/diacare.24.7.1259 11423512

[4] Nevalainen J Sairanen M Appelblom H Gissler M Timonen S Ryynänen M. First-trimester maternal serum amino acids and acylcarnitines are significant predictors of gestational diabetes. Rev. Diabetes Stud. (2016) 13:236–45. doi: 10.1900/RDS.2016.13.236 PMC5734224 28278310

[5] Piras C Neri I Pintus R Noto A Petrella E Monari F. First trimester metabolomics 1H-NMR study of the urinary profile predicts gestational diabetes mellitus development in obese women. J. Matern Fetal Neonatal Med. (2022) 35:8275–83. doi: 10.1080/14767058.2021.1970133 34530691

[6] Zhao H Li H Chung ACK Xiang L Li X Zheng Y. Large-scale longitudinal metabolomics study reveals different trimester-specific alterations of metabolites in relation to gestational diabetes mellitus. J. Proteome Res. (2019) 18:292–300. doi: 10.1021/acs.jproteome.8b00602 30488697

[7] Koos BJ Gornbein JA. Early pregnancy metabolites predict gestational diabetes mellitus: implications for fetal programming. Am. J. Obstet Gynecol. (2021) 224:215.e1–7. doi: 10.1016/j.ajog.2020.07.050 32739399

[8] Ravnsborg T Svaneklink S Andersen LLT Larsen MR Jensen DM Overgaard M. First-trimester proteomic profiling identifies novel predictors of gestational diabetes mellitus. PloS One. (2019) 14:e0214457. doi: 10.1371/journal.pone.0214457 30917176 PMC6436752

[9] Shaarbaf Eidgahi E Nasiri M Kariman N Safavi Ardebili N Salehi M Kazemi M. Diagnostic accuracy of first and early second trimester multiple biomarkers for prediction of gestational diabetes mellitus: a multivariate longitudinal approach. BMC Pregnancy Childbirth. (2022) 22:13. doi: 10.1186/s12884-021-04348-6 34983441 PMC8728972

[10] Mavreli D Evangelinakis N Papantoniou N Kolialexi A. Quantitative comparative proteomics reveals candidate biomarkers for the early prediction of gestational diabetes mellitus: A preliminary study. In Vivo. (2020) 34:517–25. doi: 10.21873/invivo.11803 PMC7157887 32111749

[11] Liu X Sun J Wen X Duan J Xue D Pan Y. Proteome profiling of gestational diabetes mellitus at 16-18 weeks revealed by LC-MS/MS. J. Clin. Lab. Anal. (2020) 34:e23424. doi: 10.1002/jcla.23424 32537767 PMC7521232

[12] Zhao C Wang F Wang P Ding H Huang X Shi Z. Early second-trimester plasma protein profiling using multiplexed isobaric tandem mass tag (TMT) labeling predicts gestational diabetes mellitus. Acta Diabetol. (2015) 52:1103–12. doi: 10.1007/s00592-015-0796-y 26259496

[13] Sharma AK Singh S Singh H Mahajan D Kolli P Mandadapu G. Deep insight of the pathophysiology of gestational diabetes mellitus. Cells. (2022) 11:2672. doi: 10.3390/cells11172672 36078079 PMC9455072

[14] Lavery JA Friedman AM Keyes KM Wright JD Ananth CV. Gestational diabetes in the United States: temporal changes in prevalence rates between 1979 and 2010. BJOG. (2017) 124:804–13. doi: 10.1111/1471-0528.14236 PMC5303559 27510598

[15] DeSisto CL Kim SY Sharma AJ. Prevalence estimates of gestational diabetes mellitus in the United States, Pregnancy Risk Assessment Monitoring System (PRAMS), 2007-2010. Prev. Chronic Dis. (2014) 11:E104. doi: 10.5888/pcd11.130415 24945238 PMC4068111

[16] Al-Rifai RH Abdo NM Paulo MS Saha S Ahmed LA. Prevalence of gestational diabetes mellitus in the middle east and north africa, 2000-2019: A systematic review, meta-analysis, and meta-regression. Front. Endocrinol. (Lausanne). (2021) 12:668447. doi: 10.3389/fendo.2021.668447 34512543 PMC8427302

[17] Getahun D Nath C Ananth CV Chavez MR Smulian JC. Gestational diabetes in the United States: temporal trends 1989 through 2004. Am. J. Obstet Gynecol. (2008) 198:525.e1–5. doi: 10.1016/j.ajog.2007.11.017 18279822

[18] Albrecht SS Kuklina EV Bansil P Jamieson DJ Whiteman MK Kourtis AP. Diabetes trends among delivery hospitalizations in the U.S., 1994-2004. Diabetes Care. (2010) 33:768–73. doi: 10.2337/dc09-1801 PMC2845025 20067968

[19] Kim SY Saraiva C Curtis M Wilson HG Troyan J Sharma AJ. Fraction of gestational diabetes mellitus attributable to overweight and obesity by race/ethnicity, California, 2007-2009. Am. J. Public Health. (2013) 103:e65–72. doi: 10.2105/AJPH.2013.301469 PMC3780749 23947320

[20] Feig DS Hwee J Shah BR Booth GL Bierman AS Lipscombe LL. Trends in incidence of diabetes in pregnancy and serious perinatal outcomes: a large, population-based study in Ontario, Canada, 1996-2010. Diabetes Care. (2014) 37:1590–6. doi: 10.2337/dc13-2717 24705609

[21] Abouzeid M Versace VL Janus ED Davey MA Philpot B Oats J. A population-based observational study of diabetes during pregnancy in Victoria, Australia, 1999-2008. BMJ Open. (2014) 4:e005394. doi: 10.1136/bmjopen-2014-005394 PMC4244457 25398676

[22] Shah NS Wang MC Freaney PM Perak AM Carnethon MR Kandula NR. Trends in gestational diabetes at first live birth by race and ethnicity in the US, 2011-2019. JAMA. (2021) 326:660–9. doi: 10.1001/jama.2021.7217 PMC8371572 34402831

[23] Kim SY England L Wilson HG Bish C Satten GA Dietz P. Percentage of gestational diabetes mellitus attributable to overweight and obesity. Am. J. Public Health. (2010) 100:1047–52. doi: 10.2105/AJPH.2009.172890 PMC2866592 20395581

[24] Di Cianni G Miccoli R Volpe L Lencioni C Del Prato S. Intermediate metabolism in normal pregnancy and in gestational diabetes. Diabetes Metab. Res. Rev. (2003) 19:259–70. doi: 10.1002/dmrr.390 12879403

[25] Catalano PM Tyzbir ED Roman NM Amini SB Sims EA. Longitudinal changes in insulin release and insulin resistance in nonobese pregnant women. Am. J. Obstet Gynecol. (1991) 165:1667–72. doi: 10.1016/0002-9378(91)90012-g 1750458

[26] Plows JF Stanley JL Baker PN Reynolds CM Vickers MH. The pathophysiology of gestational diabetes mellitus. Int. J. Mol. Sci. (2018) 19:3342. doi: 10.3390/ijms19113342 30373146 PMC6274679

[27] Lowe WL Jr Scholtens DM Sandler V Hayes MG. Genetics of gestational diabetes mellitus and maternal metabolism. Curr. Diabetes Rep. (2016) 16:15. doi: 10.1007/s11892-015-0709-z 26803651

[28] Ashcroft FM Rohm M Clark A Brereton MF. Is type 2 diabetes a glycogen storage disease of pancreatic β Cells? Cell Metab. (2017) 26:17–23. doi: 10.1016/j.cmet.2017.05.014 28683284 PMC5890904

[29] Rahier J Guiot Y Goebbels RM Sempoux C Henquin JC. Pancreatic beta-cell mass in European subjects with type 2 diabetes. Diabetes Obes. Metab. (2008) 10 Suppl 4:32–42. doi: 10.1111/j.1463-1326.2008.00969.x 18834431

[30] Van Assche FA Aerts L De Prins F. A morphological study of the endocrine pancreas in human pregnancy. Br. J. Obstet Gynaecol. (1978) 85:818–20. doi: 10.1111/j.1471-0528.1978.tb15835.x 363135

[31] Catalano PM. Trying to understand gestational diabetes. Diabetes Med. (2014) 31:273–81. doi: 10.1111/dme.12381 PMC4178541 24341419

[32] Barbour LA McCurdy CE Hernandez TL Kirwan JP Catalano PM Friedman JE. Cellular mechanisms for insulin resistance in normal pregnancy and gestational diabetes. Diabetes Care. (2007) 30 Suppl 2:S112–9. doi: 10.2337/dc07-s202 17596458

[33] Friedman JE Kirwan JP Jing M Presley L Catalano PM. Increased skeletal muscle tumor necrosis factor-alpha and impaired insulin signaling persist in obese women with gestational diabetes mellitus 1 year postpartum. Diabetes. (2008) 57:606–13. doi: 10.2337/db07-1356 PMC4697130 18083784

[34] Sivan E Boden G. Free fatty acids, insulin resistance, and pregnancy. Curr. Diabetes Rep. (2003) 3:319–22. doi: 10.1007/s11892-003-0024-y 12866995

[35] Hamilton BS Paglia D Kwan AY Deitel M. Increased obese mRNA expression in omental fat cells from massively obese humans. Nat. Med. (1995) 1:953–6. doi: 10.1038/nm0995-953 7585224

[36] Koch CE Lowe C Pretz D Steger J Williams LM Tups A. High-fat diet induces leptin resistance in leptin-deficient mice. J. Neuroendocrinol. (2014) 26:58–67. doi: 10.1111/jne.12131 24382295

[37] Honnorat D Disse E Millot L Mathiotte E Claret M Charrie A. Are third-trimester adipokines associated with higher metabolic risk among women with gestational diabetes? Diabetes Metab. (2015) 41:393–400. doi: 10.1016/j.diabet.2015.03.003 25890778

[38] Maple-Brown L Ye C Hanley AJ Connelly PW Sermer M Zinman B. Maternal pregravid weight is the primary determinant of serum leptin and its metabolic associations in pregnancy, irrespective of gestational glucose tolerance status. J. Clin. Endocrinol. Metab. (2012) 97:4148–55. doi: 10.1210/jc.2012-2290 22948759

[39] Masuzaki H Ogawa Y Sagawa N Hosoda K Matsumoto T Mise H. Nonadipose tissue production of leptin: leptin as a novel placenta-derived hormone in humans. Nat. Med. (1997) 3:1029–33. doi: 10.1038/nm0997-1029 9288733

[40] Pérez-Pérez A Maymó JL Gambino YP Guadix P Dueñas JL Varone CL. Activated translation signaling in placenta from pregnant women with gestational diabetes mellitus: possible role of leptin. Horm. Metab. Res. (2013) 45:436–42. doi: 10.1055/s-0032-1333276 23386416

[41] Williams MA Qiu C Muy-Rivera M Vadachkoria S Song T Luthy DA. Plasma adiponectin concentrations in early pregnancy and subsequent risk of gestational diabetes mellitus. J. Clin. Endocrinol. Metab. (2004) 89:2306–11. doi: 10.1210/jc.2003-031201 15126557

[42] Retnakaran R Hanley AJ Raif N Connelly PW Sermer M Zinman B. Reduced adiponectin concentration in women with gestational diabetes: a potential factor in progression to type 2 diabetes. Diabetes Care. (2004) 27:799–800. doi: 10.2337/diacare.27.3.799 14988306

[43] Succurro E Marini MA Frontoni S Hribal ML Andreozzi F Lauro R. Insulin secretion in metabolically obese, but normal weight, and in metabolically healthy but obese individuals. Obes. (Silver Spring). (2008) 16:1881–6. doi: 10.1038/oby.2008.308 18551117

[44] Wajchenberg BL. Subcutaneous and visceral adipose tissue: their relation to the metabolic syndrome. Endocr. Rev. (2000) 21:697–738. doi: 10.1210/edrv.21.6.0415 11133069

[45] Rojas-Rodriguez R Lifshitz LM Bellve KD Min SY Pires J Leung K. Human adipose tissue expansion in pregnancy is impaired in gestational diabetes mellitus. Diabetologia. (2015) 58:2106–14. doi: 10.1007/s00125-015-3662-0 PMC4526585 26067361

[46] Kautzky-Willer A Krssak M Winzer C Pacini G Tura A Farhan S. Increased intramyocellular lipid concentration identifies impaired glucose metabolism in women with previous gestational diabetes. Diabetes. (2003) 52:244–51. doi: 10.2337/diabetes.52.2.244 12540593

[47] Forbes S Taylor-Robinson SD Patel N Allan P Walker BR Johnston DG. Increased prevalence of non-alcoholic fatty liver disease in European women with a history of gestational diabetes. Diabetologia. (2011) 54:641–7. doi: 10.1007/s00125-010-2009-0 21153530

[48] Atègbo JM Grissa O Yessoufou A Hichami A Dramane KL Moutairou K. Modulation of adipokines and cytokines in gestational diabetes and macrosomia. J. Clin. Endocrinol. Metab. (2006) 91:4137–43. doi: 10.1210/jc.2006-0980 16849405

[49] Kirwan JP Hauguel-De-Mouzon S Lepercq J Challier JC Huston-Presley L Friedman JE. TNF-alpha is a predictor of insulin resistance in human pregnancy. Diabetes. (2002) 51:2207–13. doi: 10.2337/diabetes.51.7.2207 12086951

[50] Radaelli T Varastehpour A Catalano P Hauguel-de-Mouzon S. Gestational diabetes induces placental genes for chronic stress and inflammatory pathways. Diabetes. (2003) 52:2951–8. doi: 10.2337/diabetes.52.12.2951 14633856

[51] Stefan N Kantartzis K Machann J Schick F Thamer C Rittig K. Identification and characterization of metabolically benign obesity in humans. Arch. Intern. Med. (2008) 168:1609–16. doi: 10.1001/archinte.168.15.1609 18695074

[52] Zhu C Yang H Geng Q Ma Q Long Y Zhou C. Association of oxidative stress biomarkers with gestational diabetes mellitus in pregnant women: a case-control study. PloS One. (2015) 10:e0126490. doi: 10.1371/journal.pone.0126490 25915047 PMC4411158

[53] Pessler D Rudich A Bashan N. Oxidative stress impairs nuclear proteins binding to the insulin responsive element in the GLUT4 promoter. Diabetologia. (2001) 44:2156–64. doi: 10.1007/s001250100024 11793016

[54] Manea A Tanase LI Raicu M Simionescu M. Transcriptional regulation of NADPH oxidase isoforms, Nox1 and Nox4, by nuclear factor-kappaB in human aortic smooth muscle cells. Biochem. Biophys. Res. Commun. (2010) 396:901–7. doi: 10.1016/j.bbrc.2010.05.019 20457132

[55] Augustin R. The protein family of glucose transport facilitators: It’s not only about glucose after all. IUBMB Life. (2010) 62:315–33. doi: 10.1002/iub.315 20209635

[56] Hiden U Maier A Bilban M Ghaffari-Tabrizi N Wadsack C Lang I. Insulin control of placental gene expression shifts from mother to foetus over the course of pregnancy. Diabetologia. (2006) 49:123–31. doi: 10.1007/s00125-005-0054-x 16344925

[57] Jansson T Powell TL. Role of the placenta in fetal programming: underlying mechanisms and potential interventional approaches. Clin. Sci. (Lond). (2007) 113:1–13. doi: 10.1042/CS20060339 17536998

[58] Radaelli T Lepercq J Varastehpour A Basu S Catalano PM Hauguel-De-Mouzon S. Differential regulation of genes for fetoplacental lipid pathways in pregnancy with gestational and type 1 diabetes mellitus. Am. J. Obstet Gynecol. (2009) 201:209.e1–209.e10. doi: 10.1016/j.ajog.2009.04.019 PMC3613858 19560108

[59] Rinninella E Raoul P Cintoni M Franceschi F Miggiano GAD Gasbarrini A. What is the healthy gut microbiota composition? A changing ecosystem across age, environment, diet, and diseases. Microorganisms. (2019) 7:14. doi: 10.3390/microorganisms7010014 30634578 PMC6351938

[60] Arumugam M Raes J Pelletier E Le Paslier D Yamada T Mende DR. Enterotypes of the human gut microbiome. Nature. (2011) 473:174–80. doi: 10.1038/nature09944 PMC3728647 21508958

[61] Koren O Goodrich JK Cullender TC Spor A Laitinen K Bäckhed HK. Host remodeling of the gut microbiome and metabolic changes during pregnancy. Cell. (2012) 150:470–80. doi: 10.1016/j.cell.2012.07.008 PMC3505857 22863002

[62] Jin M Li D Ji R Liu W Xu X Li Y. Changes in intestinal microflora in digestive tract diseases during pregnancy. Arch. Gynecol Obstet. (2020) 301:243–9. doi: 10.1007/s00404-019-05336-0 PMC7028802 31776707

[63] Nielsen DS Møller PL Rosenfeldt V Paerregaard A Michaelsen KF Jakobsen M. Case study of the distribution of mucosa-associated Bifidobacterium species, Lactobacillus species, and other lactic acid bacteria in the human colon. Appl. Environ. Microbiol. (2003) 69:7545–8. doi: 10.1128/AEM.69.12.7545-7548.2003 PMC309914 14660412

[64] Oliphant K Allen-Vercoe E. Macronutrient metabolism by the human gut microbiome: major fermentation by-products and their impact on host health. Microbiome. (2019) 7:91. doi: 10.1186/s40168-019-0704-8 31196177 PMC6567490

[65] Kuang YS Lu JH Li SH Li JH Yuan MY He JR. Connections between the human gut microbiome and gestational diabetes mellitus. Gigascience. (2017) 6:1–12. doi: 10.1093/gigascience/gix058 PMC5597849 28873967

[66] Zhang Y Chen T Zhang Y Hu Q Wang X Chang H. Contribution of trace element exposure to gestational diabetes mellitus through disturbing the gut microbiome. Environ. Int. (2021) 153:106520. doi: 10.1016/j.envint.2021.106520 33774496 PMC8638703

[67] Ding Q Hu Y Fu Y Qian L. Systematic review and meta-analysis of the correlation between intestinal flora and gestational diabetes mellitus. Ann. Palliat Med. (2021) 10:9752–64. doi: 10.21037/apm-21-2061 34628901

[68] Rafat D Singh S Nawab T Khan F Khan AU Khalid S. Association of vaginal dysbiosis and gestational diabetes mellitus with adverse perinatal outcomes. Int. J. Gynaecol Obstet. (2022) 158:70–8. doi: 10.1002/ijgo.13945 34561861

[69] Cortez RV Taddei CR Sparvoli LG Ângelo AGS Padilha M Mattar R. Microbiome and its relation to gestational diabetes. Endocrine. (2019) 64:254–64. doi: 10.1007/s12020-018-1813-z 30421135

[70] Han M-M Sun J-F Su X-H Peng YF Goyal H Wu CH. Probiotics improve glucose and lipid metabolism in pregnant women: a meta-analysis. Ann. Transl. Med. (2019) 7:99–9. doi: 10.21037/atm.2019.01.61 PMC6462661 31019949

[71] Dallanora S Medeiros de Souza Y Deon RG Tracey CA Freitas-Vilela AA Wurdig Roesch LF. Do probiotics effectively ameliorate glycemic control during gestational diabetes? A systematic review. Arch. Gynecol Obstet. (2018) 298:477–85. doi: 10.1007/s00404-018-4809-2 29916111

[72] Taylor BL Woodfall GE Sheedy KE O'Riley ML Rainbow KA Bramwell EL. Effect of probiotics on metabolic outcomes in pregnant women with gestational diabetes: A systematic review and meta-analysis of randomized controlled trials. Nutrients. (2017) 9:461. doi: 10.3390/nu9050461 28475161 PMC5452191

[73] Jafarnejad S Saremi S Jafarnejad F Arab A. Effects of a multispecies probiotic mixture on glycemic control and inflammatory status in women with gestational diabetes: A randomized controlled clinical trial. J. Nutr. Metab. (2016) 2016. doi: 10.1155/2016/5190846 PMC4939193 27429803

[74] Sahhaf Ebrahimi F Homayouni Rad A Mosen M Abbasalizadeh F Tabrizi A Khalili L. Effect of L. acidophilus and B. lactis on blood glucose in women with gestational diabetes mellitus: A randomized placebo-controlled trial. Diabetol. Metab. Syndrome. (2019) 11:1–7. doi: 10.1186/s13098-019-0471-5 PMC6714347 31485272

[75] Okesene-Gafa KAM Moore AE Jordan V McCowan L Crowther CA. Probiotic treatment for women with gestational diabetes to improve maternal and infant health and well-being. Cochrane Database Systematic Rev. (2020) 2020. doi: 10.1002/14651858.CD012970.pub2 PMC7386668 32575163

[76] Luoto R Laitinen K Nermes M Isolauri E. Impact of maternal probiotic-supplemented dietary counselling on pregnancy outcome and prenatal and postnatal growth: a double-blind, placebo-controlled study. Br. J. Nutr. (2010) 103:1792–9. doi: 10.1017/S0007114509993898 20128938

[77] Asemi Z Samimi M Tabassi Z Rad MN Foroushani AR Khorammian H. Effect of daily consumption of probiotic yoghurt on insulin resistance in pregnant women: a randomized controlled trial. Eur. J. Clin. Nutr. (2013) 67:71. doi: 10.1038/ejcn.2012.189 23187955

[78] Karamali M Dadkhah F Sadrkhanlou M Jamilian M Ahmadi S Tajabadi-Ebrahimi M. Effects of probiotic supplementation on glycaemic control and lipid profiles in gestational diabetes: A randomized, double-blind, placebo-controlled trial. Diabetes Metab. (2016) 42:234–41. doi: 10.1016/j.diabet.2016.04.009 27209439

[79] Dolatkhah N Hajifaraji M Abbasalizadeh F Aghamohammadzadeh N Mehrabi Y Abbasi MM. Is there a value for probiotic supplements in gestational diabetes mellitus? A randomized clinical trial. J. Health Popul Nutr. (2015) 33:25. doi: 10.1186/s41043-015-0034-9 26825666 PMC5026018

[80] Yefet E Bar L Izhaki I Iskander R Massalha M Younis JS. Effects of probiotics on glycemic control and metabolic parameters in gestational diabetes mellitus: systematic review and meta-analysis. Nutrients. (2023) 15:1633. doi: 10.3390/nu15071633 37049473 PMC10097303

[81] de Veciana M Major CA Morgan MA Asrat T Toohey JS Lien JM. Postprandial versus preprandial blood glucose monitoring in women with gestational diabetes mellitus requiring insulin therapy. N Engl. J. Med. (1995) 333:1237–41. doi: 10.1056/NEJM199511093331901 7565999

[82] Nachum Z Perlitz Y Shavit LY Magril G Vitner D Zipori Y. The effect of oral probiotics on glycemic control of women with gestational diabetes mellitus-a multicenter, randomized, double-blind, placebo-controlled trial. Am. J. Obstet Gynecol MFM. (2024) 6:101224. doi: 10.1016/j.ajogmf.2023.101224 37956906

[83] Parrettini S Caroli A Torlone E. Nutrition and metabolic adaptations in physiological and complicated pregnancy, focus on obesity and gestational diabetes. Front. Endocrinol. (2020) 11:611929. doi: 10.3389/fendo.2020.611929 PMC7793966 33424775

[84] Hay DL Lopata A. Chorionic gonadotropin secretion by human embryos *in vitro* . J. Clin. Endocrinol. Metab. (1988) 67:1322–4. doi: 10.1210/jcem-67-6-1322 2461389

[85] Donovan BM Nidey NL Jasper EA Robinson JG Bao W Saftlas AF. First trimester prenatal screening biomarkers and gestational diabetes mellitus, A systematic review and meta-analysis. PloS One. (2018) 13:e0201319. doi: 10.1371/journal.pone.0201319 30048548 PMC6062092

[86] Valent AM Choi H Kolahi KS Thornburg KL. Hyperglycemia and gestational diabetes suppress placental glycolysis and mitochondrial function and alter lipid processing. FASEB J. (2021) 35:e21423. doi: 10.1096/fj.202000326RR 33605480 PMC8906558

[87] Frankenne F Closset J Gomez F Scippo ML Smal J Hennen G. The physiology of growth hormones (GHs) in pregnant women and partial characterization of the placental GH variant. J. Clin. Endocrinol. Metab. (1988) 66:1171–80. doi: 10.1210/jcem-66-6-1171 3372680

[88] Patel N Alsat E Igout A Baron F Hennen G Porquet D. Glucose inhibits human placental GH secretion, *in vitro* . J. Clin. Endocrinol. Metab. (1995) 80:1743–6. 10.1210/jcem.80.5.7745029 7745029

[89] Wang S Wu J Wang N Zeng L Wu Y. The role of growth hormone receptor in beta cell function. Growth Horm. IGF Res. (2017) 36:30–5. doi: 10.1016/j.ghir.2017.08.002 28915386

[90] Alsat E Guibourdenche J Luton D Frankenne F Evain-Brion D. Human placental growth hormone. Am. J. Obstet Gynecol. (1997) 177:1526–34. doi: 10.1016/S0002-9378(97)70103-0 9423763

[91] Le TN Elsea SH Romero R Chaiworapongsa T Francis GL. Prolactin receptor gene polymorphisms are associated with gestational diabetes. Genet. Test Mol. biomark. (2013) 17:567–71. doi: 10.1089/gtmb.2013.0009 PMC3700434 23651351

[92] Ngala RA Fondjo LA Gmagna P Ghartey FN Awe MA. Placental peptides metabolism and maternal factors as predictors of risk of gestational diabetes in pregnant women: A case-control study. PloS One. (2017) 12:e0181613. doi: 10.1371/journal.pone.0181613 28732072 PMC5521813

[93] Retnakaran R Ye C Kramer CK Connelly PW Hanley AJ Sermer M. Evaluation of circulating determinants of beta-cell function in women with and without gestational diabetes. J. Clin. Endocrinol. Metab. (2016) 101:2683–91. doi: 10.1210/jc.2016-1402 27023450

[94] Hauguel-de-Mouzon S Lepercq J Catalano P. The known and unknown of leptin in pregnancy. Am. J. Obstet Gynecol. (2006) 194:1537–45. doi: 10.1016/j.ajog.2005.06.064 16731069

[95] Grasman J. Reconstruction of the drive underlying food intake and its control by leptin and dieting. PloS One. (2013) 8:e74997. doi: 10.1371/journal.pone.0074997 24086420 PMC3783460

[96] Shang M Dong X Hou L. Correlation of adipokines and markers of oxidative stress in women with gestational diabetes mellitus and their newborns. J. Obstet Gynaecol Res. (2018) 44:637–46. doi: 10.1111/jog.13586 29399931

[97] Qiu C Williams MA Vadachkoria S Frederick IO Luthy DA. Increased maternal plasma leptin in early pregnancy and risk of gestational diabetes mellitus. Obstet Gynecol. (2004) 103:519–25. doi: 10.1097/01.AOG.0000113621.53602.7a 14990416

[98] Mauvais-Jarvis F. Role of sex steroids in beta cell function, growth, and survival. Trends Endocrinol. Metab. (2016) 27:844–55. doi: 10.1016/j.tem.2016.08.008 PMC5116277 27640750

[99] Hur J Cho EH Baek KH Lee KJ. Prediction of gestational diabetes mellitus by unconjugated estriol levels in maternal serum. Int. J. Med. Sci. (2017) 14:123–7. doi: 10.7150/ijms.17321 PMC5332840 28260987

[100] Kleiblova P Dostalova I Bartlova M Lacinova Z Ticha I Krejci V. Expression of adipokines and estrogen receptors in adipose tissue and placenta of patients with gestational diabetes mellitus. Mol. Cell Endocrinol. (2010) 314:150–6. doi: 10.1016/j.mce.2009.08.002 19682537

[101] Freemark M. Regulation of maternal metabolism by pituitary and placental hormones, Roles in fetal development and metabolic programming. Horm. Res. (2006) 65:41–9. doi: 10.1159/000091505 16612113

[102] Branisteanu DD Mathieu C. Progesterone in gestational diabetes mellitus, Guilty or not guilty? Trends Endocrinol. Metab. (2003) 14:54–6. doi: 10.1016/S1043-2760(03)00003-1 12591170

[103] Li M Song Y Rawal S Hinkle SN Zhu Y Tekola-Ayele F. Plasma prolactin and progesterone levels and the risk of gestational diabetes, A prospective and longitudinal study in a multiracial cohort. Front. Endocrinol. (2020) 11:83. doi: 10.3389/fendo.2020.00083 PMC7058109 32180760

[104] Zhang Z Kang X Guo Y Zhang J Xie J Shao S. Association of circulating galectin-3 with gestational diabetes mellitus, progesterone, and insulin resistance. J. Diabetes. (2021) 13:54–62. doi: 10.1111/1753-0407.13088 32671973

[105] Rebarber A Istwan NB Russo-Stieglitz K Cleary-Goldman J Rhea DJ Stanziano GJ. Increased incidence of gestational diabetes in women receiving prophylactic 17alpha-hydroxyprogesterone caproate for prevention of recurrent preterm delivery. Diabetes Care. (2007) 30:2277–80. doi: 10.2337/dc07-0564 17563346

[106] Retnakaran R Kramer CK Ye C Kew S Hanley AJ Connelly PW. Fetal sex and maternal risk of gestational diabetes mellitus, the impact of having a boy. Diabetes Care. (2015) 38:844–51. doi: 10.2337/dc14-2551 25693837

[107] Jaskolka D Retnakaran R Zinman B Kramer CK. Sex of the baby and risk of gestational diabetes mellitus in the mother, A systematic review and meta-analysis. Diabetologia. (2015) 58:2469–75. doi: 10.1007/s00125-015-3726-1 26253767

[108] Retnakaran R Shah BR. Sex of the baby and future maternal risk of Type 2 diabetes in women who had gestational diabetes. Diabetes Med. (2016) 33:956–60. doi: 10.1111/dme.12989 26470996

[109] Walsh JM Segurado R Mahony RM Foley ME McAuliffe FM. The effects of fetal gender on maternal and fetal insulin resistance. PloS One. (2015) 10:e0137215. doi: 10.1371/journal.pone.0137215 26368559 PMC4569192

[110] Yamashita H Yasuhi I Koga M Sugimi S Umezaki Y Fukuoka M. Fetal sex and maternal insulin resistance during mid-pregnancy, A retrospective cohort study. BMC Pregnancy Childbirth. (2020) 20:560. doi: 10.1186/s12884-020-03242-x 32972384 PMC7513312

[111] Rafferty AR Geraghty AA Kennelly MA O’Brien EC Reji RM Mehegan J. Limited impact of fetal sex and maternal body mass index on fetal and maternal insulin resistance and lipid metabolism, findings from the PEARs study. Reprod. Sci. (2020) 27:513–22. doi: 10.1007/s43032-019-00045-0 31925771

[112] Gol M Altunyurt S Cimrin D Guclu S Bagci M Demir N. Different maternal serum hCG levels in pregnant women with female and male fetuses, Does fetal hypophyseal-adrenal-gonadal axis play a role? J. Perinat Med. (2004) 32:342–5. doi: 10.1515/JPM.2004.064 15346821

[113] Chellakooty M Skibsted L Skouby SO Andersson AM Petersen JH Main KM. Longitudinal study of serum placental GH in 455 normal pregnancies, Correlation to gestational age, fetal gender, and weight. J. Clin. Endocrinol. Metab. (2002) 87:2734–9. doi: 10.1210/jcem.87.6.8544 12050242

[114] Eriksson JG Kajantie E Osmond C Thornburg K Barker DJ. Boys live dangerously in the womb. Am. J. Hum. Biol. (2010) 22:330–5. doi: 10.1002/ajhb.20995 PMC3923652 19844898

[115] American College of Obstetricians and Gynecologists’ Committee on Practice Bulletins—Obstetrics. Practice Bulletin No. 173: Fetal Macrosomia. Obstet Gynecol. (2016) 128(5):e195–e209. doi: 10.1097/AOG.0000000000001767 27776071

[116] Johnstone FD Prescott RJ Steel JM. Clinical and ultrasound prediction of macrosomia in diabetic pregnancy. Br. J. Obstet Gynaecol. (1996) 103:747. 8760702 10.1111/j.1471-0528.1996.tb09868.x

[117] O’Reilly-Green C Divon M. Sonographic and clinical methods in the diagnosis of macrosomia. Clin. Obstet Gynecol. (2000) 43:309. doi: 10.1097/00003081-200006000-00008 10863628

[118] Farrell T Holmes R Stone P. The effect of body mass index on three methods of fetal weight estimation. BJOG. (2002) 109:651–7. doi: 10.1111/j.1471-0528.2002.01249.x 12118643

[119] Pretscher J Kehl S Stumpfe FM Mayr A Schmid M Schild RL. Ultrasound fetal weight estimation in diabetic pregnancies. J. Ultrasound Med. (2020) 39:341–50. doi: 10.1002/jum.15112 31436342

[120] Ben-Haroush A Yogev Y Mashiach R Hod M Meisner I. Accuracy of sonographic estimation of fetal weight before induction of labor in diabetic pregnancies and pregnancies with suspected fetal macrosomia. J. Perinat Med. (2003) 31:225–30. doi: 10.1515/JPM.2003.030 12825478

[121] Shmueli A Salman L Hadar E Aviram A Bardin R Ashwal E. Sonographic prediction of macrosomia in pregnancies complicated by maternal diabetes: finding the best formula. Arch. Gynecol Obstet. (2019) 299:97–103. doi: 10.1007/s00404-018-4934-y 30327863

[122] Hadlock FP Harrist RB Sharman RS Deter RL Park SK. Estimation of fetal weight with the use of head, body, and femur measurements: a prospective study. Am. J. Obstet Gynecol. (1985) 151:333–7. doi: 10.1016/0002-9378(85)90298-4 3881966

[123] Cesnaite G Domza G Ramasauskaite D Volochovic J. The accuracy of 22 fetal weight estimation formulas in diabetic pregnancies. Fetal Diagn. Ther. (2020) 47:54–9. doi: 10.1159/000500452 31195392

[124] Hsieh FJ Chang FM Huang HC Lu CC Ko TM Chen HY. Computer-assisted analysis for prediction of fetal weight by ultrasound comparison of biparietal diameter (BPD), abdominal circumference (AC) and femur length (FL). Taiwan Yi Xue Hui Za Zhi. (1987) 86:957–64. 3320270

[125] Farrell T Fraser R Chan K. Ultrasonic fetal weight estimation in women with pregnancy complicated by diabetes. Acta Obstet Gynecol Scand. (2004) 83:1065–6. doi: 10.1111/j.0001-6349.2004.00469.x 15488123

[126] Ben-Haroush A Chen R Hadar E Hod M Yogev Y. Accuracy of a single fetal weight estimation at 29-34 weeks in diabetic pregnancies: can it predict large-for-gestational-age infants at term? Am. J. Obstet Gynecol. (2007) 197:497. doi: 10.1016/j.ajog.2007.04.023 17980186

[127] Hedriana HL Moore TR. A comparison of single versus multiple growth ultrasonographic examinations in predicting birth weight. Am. J. Obstet Gynecol. (1994) 170:1600–4. doi: 10.1016/s0002-9378(94)70329-9 8203416

[128] Lee W Balasubramaniam M Deter RL Yeo L Hassan SS Gotsch F. New fetal weight estimation models using fractional limb volume. Ultrasound Obstet Gynecol. (2009) 34:556–65. doi: 10.1002/uog.v34:5 PMC2784152 19725080

[129] Lee W Deter R Sangi-Haghpeykar H Yeo L Romero R. Prospective validation of fetal weight estimation using fractional limb volume. Ultrasound Obstet Gynecol. (2013) 41:198–203. doi: 10.1002/uog.2013.41.issue-2 22605519 PMC3601845

[130] Pagani G Palai N Zatti S Fratelli N Prefumo F Frusca T. Fetal weight estimation in gestational diabetic pregnancies: comparison between conventional and three-dimensional fractional thigh volume methods using gestation-adjusted projection. Ultrasound Obstet Gynecol. (2014) 43:72–6. doi: 10.1002/uog.12458 23494762

[131] Lee W Mack LM Gandhi R Sangi-Haghpeykar H. Fetal weight estimation using automated fractional limb volume with 2-dimensional size parameters in diabetic pregnancies. J. Ultrasound Med. (2021) 40:279–84. doi: 10.1002/jum.15397 32710582

[132] Tuuli MG Kapalka K Macones GA Cahill AG. Three-versus two-dimensional sonographic biometry for predicting birth weight and macrosomia in diabetic pregnancies. J. Ultrasound Med. (2016) 35:1925–30. doi: 10.7863/ultra.15.08032 27466257

[133] Baker PN Johnson IR Gowland PA. Fetal weight estimation by echo-planar magnetic resonance imaging. Lancet. (1994) 343:644. doi: 10.1016/S0140-6736(94)92638-7 7906814

[134] Uotila J Dastidar P Heinonen T Ryymin P Punnonen R Laasonen E. Magnetic resonance imaging compared to ultrasonography in fetal weight and volume estimation in diabetic and normal pregnancy. Acta Obstet Gynecol Scand. (2000) 79:255–9. doi: 10.1034/j.1600-0412.2000.079004255.x 10746838

[135] O’Sullivan JB Mahan CM Charles D Dandrow RV. Screening criteria for high risk gestational diabetic patients. Am. J. Obstetrics Gynecology. (1973) 116:895–900. doi: 10.1016/s0002-9378(16)33833-9 4718216

[136] Vandorsten JP Dodson WC Espeland MA Grobman WA Guise JM Mercer BM. NIH consensus development conference: diagnosing gestational diabetes mellitus. NIH Consens State Sci. Statements. (2013) 29:1–31. 23748438

[137] Esakoff TF Cheng YW Caughey AB. Screening for gestational diabetes: different cut-offs for different ethnicities? Am. J. Obstet Gynecol. (2005) 193:1040–4. doi: 10.1016/j.ajog.2005.05.084 16157108

[138] ACOG technical bulletin. Diabetes and pregnancy. Number 200–December 1994 (replaces No. 92, May 1986). Committee on Technical Bulletins of the American College of Obstetricians and Gynecologists. Int. J. Gynaecol Obstet. (1995) 48:331–9. doi: 10.1016/0020-7292(95)90312-7 7781883

[139] Moyer VA. Screening for gestational diabetes mellitus: U.S. Preventive Services Task Force recommendation statement. U.S. Preventive Services Task Force. Ann. Intern. Med. (2014) 160:414–20. doi: 10.7326/M13-2905 24424622

[140] American Diabetes Association. 2. Classification and Diagnosis of Diabetes. Diabetes Care. (2017) 40:S11–24. doi: 10.2337/dc17-S005 27979889

[141] Amylidi S Mosimann B Stettler C Fiedler GM Surbek D Raio L. First-trimester glycosylated hemoglobin in women at high risk for gestational diabetes. Acta Obstet Gynecol Scand. (2016) 95:93–7. doi: 10.1111/aogs.2016.95.issue-1 26400192

[142] Yefet E Jeda E Yossef A Massalha M Tzur A Nachum Z. Risk for fetal malformations and unfavorable neonatal outcomes in early-onset gestational diabetes mellitus. J. Endocrinol. Invest. (2023). doi: 10.1007/s40618-023-02238-6 38042766

[143] American Diabetes Association. Management of diabetes in pregnancy. Diabetes Care. (2017) 40:S114–9. doi: 10.2337/dc17-S016

[144] Yefet E Jeda E Tzur A Nachum Z. Markers for undiagnosed type 2 diabetes mellitus during pregnancy-A population-based retrospective cohort study. J Diabetes. (2020) 12(3):205–14. doi: 10.1111/1753-0407.12985 31498952

[145] Classification and diagnosis of diabetes mellitus and other categories of glucose intolerance. National Diabetes Data Group. Diabetes. (1979) 28:1039–57. doi: 10.2337/diab.28.12.1039 510803

[146] Carpenter MW Coustan DR. Criteria for screening tests for gestational diabetes. Am. J. Obstet Gynecol. (1982) 144:768–73. doi: 10.1016/0002-9378(82)90349-0 7148898

[147] Landon MB Spong CY Thom E Carpenter MW Ramin SM Casey B. A multicenter, randomized trial of treatment for mild gestational diabetes. N Engl J Med. (2009) 361:1339–48. doi: 10.1056/NEJMoa0902430 PMC2804874 19797280

[148] HAPO Study Cooperative Research Group Metzger BE Lowe LP Dyer AR Trimble ER Chaovarindr U. Hyperglycemia and adverse pregnancy outcomes. N Engl. J. Med. (2008) 358:1991–2002. 18463375 10.1056/NEJMoa0707943

[149] Bodmer-Roy S Morin L Cousineau J Rey E. Pregnancy outcomes in women with and without gestational diabetes mellitus according to the International Association of the Diabetes and Pregnancy Study Groups criteria. Obstet Gynecol. (2012) 120:746–52. doi: 10.1097/AOG.0b013e31826994ec 22996090

[150] Saccone G Khalifeh A Al-Kouatly HB Sendek K Berghella V. Screening for gestational diabetes mellitus: one step versus two step approach. A meta-analysis of randomized trials. J. Matern Fetal Neonatal Med. (2020) 33:1616–24. doi: 10.1080/14767058.2018.1519543 30173594

[151] Hillier TA Pedula KL Ogasawara KK Vesco KK Oshiro CES Lubarsky SL. A pragmatic, randomized clinical trial of gestational diabetes screening. N Engl. J. Med. (2021) 384:895–904. doi: 10.1056/NEJMoa2026028 33704936 PMC9041326

[152] Davis EM Abebe KZ Simhan HN Catalano P Costacou T Comer D. Perinatal outcomes of two screening strategies for gestational diabetes mellitus: A randomized controlled trial. Obstet Gynecol. (2021) 138:6–15. doi: 10.1097/AOG.0000000000004431 34259458 PMC8288467

[153] Marchetti D Carrozzino D Fraticelli F Fulcheri M Vitacolonna E. Quality of life in women with gestational diabetes mellitus: A systematic review. J. Diabetes Res. (2017) 2017:7058082. doi: 10.1155/2017/7058082 28326332 PMC5343261

[154] Craig L Sims R Glasziou P Thomas R. Women’s experiences of a diagnosis of gestational diabetes mellitus: a systematic review. BMC Pregnancy Childbirth. (2020) 20:76. doi: 10.1186/s12884-020-2745-1 32028931 PMC7006162

[155] Cheng YW Block-Kurbisch I Caughey AB. Carpenter-Coustan criteria compared with the national diabetes data group thresholds for gestational diabetes mellitus. Obstet Gynecol. (2009). doi: 10.1097/AOG.0b013e3181ae8d85 19622994

[156] Roeckner JT Sanchez-Ramos L Jijon-Knupp R Kaunitz AM. Single abnormal value on 3-hour oral glucose tolerance test during pregnancy is associated with adverse maternal and neonatal outcomes: a systematic review and metaanalysis. Am. J. Obstet Gynecol. (2016) 215:287–97. doi: 10.1016/j.ajog.2016.04.040 27133007

[157] Langer O Anyaegbunam A Brustman L Divon M. Management of women with one abnormal oral glucose tolerance test value reduces adverse outcome in pregnancy. Am. J. Obstet Gynecol. (1989) 161:593–9. doi: 10.1016/0002-9378(89)90361-x 2675597

[158] ACOG practice bulletin no. 190: gestational diabetes mellitus. Obstet Gynecol. (2018) 131:e49. doi: 10.1097/AOG.0000000000002501 29370047

[159] Raman P Shepherd E Dowswell T Middleton P Crowther CA. Different methods and settings for glucose monitoring for gestational diabetes during pregnancy. Cochrane Database Syst. Rev. (2017) 10. doi: 10.1002/14651858.CD011069.pub2 PMC6485695 29081069

[160] Weisz B Shrim A Homko CJ Schiff E Epstein GS Sivan E. One hour versus two hours postprandial glucose measurement in gestational diabetes: a prospective study. J. Perinatol. (2005) 25:241–4. doi: 10.1038/sj.jp.7211243 15605070

[161] Moses RG Lucas EM Knights S. Gestational diabetes mellitus. At what time should the postprandial glucose level be monitored? Aust. N Z J. Obstet Gynaecol. (1999) 39:457–60. doi: 10.1111/j.1479-828x.1999.tb03132.x 10687763

[162] Sivan E Weisz B Homko CJ Reece EA Schiff E. One or two hours postprandial glucose measurements: are they the same? Am. J. Obstet Gynecol. (2001) 185:604–7. doi: 10.1067/mob.2001.117184 11568785

[163] Ben-Haroush A Yogev Y Chen R Rosenn B Hod M Langer O. The postprandial glucose profile in the diabetic pregnancy. Am. J. Obstet Gynecol. (2004) 191:576–81. doi: 10.1016/j.ajog.2004.01.055 15343240

[164] Nachum Z Ben-Shlomo I Weiner E Ben-Ami M Shalev E. Diabetes in pregnancy: efficacy and cost of hospitalization as compared with ambulatory management–a prospective controlled study. Isr. Med. Assoc. J. (2001) 3:915–9. 11794914

[165] Crowther CA Hiller JE Moss JR McPhee AJ Jeffries WS Robinson JS. Effect of treatment of gestational diabetes mellitus on pregnancy outcomes. N Engl. J. Med. (2005) 352:2477–86. doi: 10.1056/NEJMoa042973 15951574

[166] Simmons D Immanuel J Hague WM Teede H Nolan CJ Peek MJ. Treatment of gestational diabetes mellitus diagnosed early in pregnancy. N Engl J Med. (2023) 388:2132–44. doi: 10.1056/NEJMoa2214956 37144983

[167] Langer O Rodriguez DA Xenakis EM McFarland MB Berkus MD Arrendondo F. Intensified versus conventional management of gestational diabetes. Am J Obstet Gynecol. (1994) 170:1036–46. doi: 10.1016/S0002-9378(94)70097-4 8166187

[168] Nachum Z Ben-Shlomo I Weiner E Shalev E. Twice daily versus four times daily insulin dose regimens for diabetes in pregnancy: randomised controlled trial. BMJ. (1999) 319:1223–7. doi: 10.1136/bmj.319.7219.1223 PMC28269 10550081

[169] Brown J Alwan NA West J Brown S McKinlay CJ Farrar D. Lifestyle interventions for the treatment of women with gestational diabetes. Cochrane Database Systematic Rev. (2017). doi: 10.1002/14651858.CD011970.pub2 PMC6481373 28472859

[170] Langer O Conway DL Berkus MD Xenakis EM Gonzales O. A comparison of glyburide and insulin in women with gestational diabetes mellitus. N Engl. J. Med. (2000) 343:1134–8. doi: 10.1056/NEJM200010193431601 11036118

[171] Elliott BD Langer O Schenker S Johnson RF. Insignificant transfer of glyburide occurs across the human placenta. Am. J. Obstet Gynecol. (1991) 165:807–12. doi: 10.1016/0002-9378(91)90421-m 1951536

[172] Elliott BD Schenker S Langer O Johnson R Prihoda T. Comparative placental transport of oral hypoglycemic agents in humans: a model of human placental drug transfer. Am. J. Obstet Gynecol. (1994) 171:653–60. doi: 10.1016/0002-9378(94)90078-7 8092211

[173] Eyal S Easterling TR Carr D Umans JG Miodovnik M Hankins GD. Pharmacokinetics of metformin during pregnancy. Drug Metab. Dispos. (2010) 38:833–40. doi: 10.1124/dmd.109.031245 PMC2872944 20118196

[174] Gilbert C Valois M Koren G. Pregnancy outcome after first-trimester exposure to metformin: a meta-analysis. Fertil Steril. (2006) 86:658–63. doi: 10.1016/j.fertnstert.2006.02.098 16879826

[175] Gargaun S Ryan E Greenblatt E Fettes I Shapiro H Padjen A. Pregnancy outcome in women with polycystic ovary syndrome exposed to metformin. Can. J. Clin. Pharmacol. (2003) 10:e149.

[176] Zhuo Z Wang A Yu H. Effect of metformin intervention during pregnancy on the gestational diabetes mellitus in women with polycystic ovary syndrome: a systematic review and meta-analysis. J. Diabetes Res. (2014) 2014:381231. doi: 10.1155/2014/381231 24963493 PMC4055053

[177] Kjerpeseth LJ Cesta CE Furu K Engeland A Gissler M Gulseth HL. Metformin versus insulin and risk of major congenital malformations in pregnancies with type 2 diabetes: A nordic register-based cohort study. Diabetes Care 1 August. (2023) 46:1556–64. doi: 10.2337/dc23-0256 37343541

[178] Rowan JA Hague WM Gao W Battin MR Moore MP. Metformin versus insulin for the treatment of gestational diabetes. N Engl. J. Med. (2008) 358:2003–15. doi: 10.1056/NEJMoa0707193 18463376

[179] Spaulonci CP Bernardes LS Trindade TC Zugaib M Francisco RP. Randomized trial of metformin vs insulin in the management of gestational diabetes. Am. J. Obstet Gynecol. (2013) 209:34.e1–7. doi: 10.1016/j.ajog.2013.03.022 23524173

[180] Picón-César MJ Molina-Vega M Suárez-Arana M González-Mesa E Sola-Moyano AP Roldan-López R. Metformin for gestational diabetes study: metformin vs insulin in gestational diabetes: glycemic control and obstetrical and perinatal outcomes: randomized prospective trial. Am. J. Obstet Gynecol. (2021) 225:517.e1–517.e17. doi: 10.1016/j.ajog.2021.04.229 33887240

[181] Balsells M García-Patterson A Solà I Roqué M Gich I Corcoy R. Glibenclamide, metformin, and insulin for the treatment of gestational diabetes: a systematic review and meta-analysis. BMJ. (2015) 350:h102. doi: 10.1136/bmj.h102 25609400 PMC4301599

[182] Camelo Castillo W Boggess K Stürmer T Brookhart MA Benjamin Jr Jonsson Funk DK M. Association of adverse pregnancy outcomes with glyburide versus insulin in women with gestational diabetes. JAMA Pediatr. (2015) 169(5):452–8. doi: 10.1001/jamapediatrics.2015.74 25822253

[183] Nicholson WK Wilson LM Witkop CT Baptiste-Roberts K Bennett WL Bolen S. Therapeutic management, delivery, and postpartum risk assessment and screening in gestational diabetes. Evid Rep. Technol. Assess. (Full Rep). (2008) 162):1–96. PMC4781072 18457474

[184] Nicholson W Bolen S Witkop CT Neale D Wilson L Bass E. Benefits and risks of oral diabetes agents compared with insulin in women with gestational diabetes: a systematic review. Obstet Gynecol. (2009) 113:193–205. doi: 10.1097/AOG.0b013e318190a459 19104375

[185] Dhulkotia JS Ola B Fraser R Farrell T. Oral hypoglycemic agents vs insulin in management of gestational diabetes: a systematic review and metaanalysis. Am. J. Obstet Gynecol. (2010) 203:457.e1–9. doi: 10.1016/j.ajog.2010.06.044 20739011

[186] Brown J Grzeskowiak L Williamson K Downie MR Crowther CA. Insulin for the treatment of women with gestational diabetes. Cochrane Database Syst. Rev. (2017) 11:CD012037. doi: 10.1002/14651858.CD012037.pub2 29103210 PMC6486160

[187] Tarry-Adkins JL Aiken CE Ozanne SE. Comparative impact of pharmacological treatments for gestational diabetes on neonatal anthropometry independent of maternal glycaemic control: A systematic review and meta-analysis. PloS Med. (2020) 17:e1003126. doi: 10.1371/journal.pmed.1003126 32442232 PMC7244100

[188] Butalia S Gutierrez L Lodha A Aitken E Zakariasen A Donovan L. Short- and long-term outcomes of metformin compared with insulin alone in pregnancy: a systematic review and meta-analysis. Diabetes Med. (2017) 34:27–36. doi: 10.1111/dme.13150 27150509

[189] Brown J Martis R Hughes B Rowan J Crowther CA. Oral anti-diabetic pharmacological therapies for the treatment of women with gestational diabetes. Cochrane Database Syst. Rev. (2017) 1:CD011967. doi: 10.1002/14651858.CD011967.pub2 28120427 PMC6464763

[190] Nachum Z Zafran N Salim R Hissin N Hasanein J Gam Ze Letova Y. Glyburide versus metformin and their combination for the treatment of gestational diabetes mellitus: A randomized controlled study. Diabetes Care. (2017) 40:332–7. doi: 10.2337/dc16-2307 28077460

[191] Wouldes TA Battin M Coat S Rush EC Hague WM Rowan JA. Neurodevelopmental outcome at 2 years in offspring of women randomised to metformin or insulin treatment for gestational diabetes. Arch. Dis. Child Fetal Neonatal Ed. (2016) 101:F488–93. doi: 10.1136/archdischild-2015-309602 26912348

[192] Gordon HG Atkinson JA Tong S Mehdipour P Cluver C Walker SP. Metformin in pregnancy and childhood neurodevelopmental outcomes: a systematic review and meta-analysis. Am J Obstet Gynecol. (2024) 231(3):308–14.e6. doi: 10.1016/j.ajog.2024.02.316 38460832

[193] Kimber-Trojnar Z Marciniak B Leszczyńska-Gorzelak B Trojnar M Oleszczuk J. Glyburide for the treatment of gestational diabetes mellitus. Pharmacol. Rep. (2008) 60:308–18. 18622055

[194] Schwartz RA Rosenn B Aleksa K Koren G. Glyburide transport across the human placenta. Obstet Gynecol. (2015) 125:583–8. doi: 10.1097/AOG.0000000000000672 25730219

[195] Hebert MF Ma X Naraharisetti SB Obstetric-Fetal Pharmacology Research Unit Network. Are we optimizing gestational diabetes treatment with glyburide? The pharmacologic basis for better clinical practice. Clin. Pharmacol. Ther. (2009) 85:607–14. doi: 10.1038/clpt.2009.5 PMC2684566 19295505

[196] Venkatesh KK Chiang CW Castillo WC Battarbee AN Donneyong M Harper LM. Changing patterns in medication prescription for gestational diabetes during a time of guideline change in the USA: a cross-sectional study. BJOG. (2022) 129(3):473–83. doi: 10.1111/1471-0528.16960 PMC8752504 34605130

[197] Society of Maternal-Fetal Medicine (SMFM) Publications Committee. SMFM Statement: Pharmacological treatment of gestational diabetes. Am. J. Obstet Gynecol. (2018) 218:B2–4. doi: 10.1016/j.ajog.2018.01.041 29409848

[198] Foster-Powell KA Cheung NW. Recurrence of gestational diabetes. Aust. N Z J. Obstet Gynaecol. (1998) 38:384–7. doi: 10.1111/j.1479-828x.1998.tb03092.x 9890214

[199] Spong CY Guillermo L Kuboshige J Cabalum T. Recurrence of gestational diabetes mellitus: identification of risk factors. Am. J. Perinatol. (1998) 15:29–33. doi: 10.1055/s-2007-993894 9475684

[200] Major CA deVeciana M Weeks J Morgan MA. Recurrence of gestational diabetes: who is at risk? Am. J. Obstet Gynecol. (1998) 179:1038–42. doi: 10.1016/S0002-9378(98)70211-X 9790394

[201] Nohira T Kim S Nakai H Okabe K Nohira T Yoneyama K. Recurrence of gestational diabetes mellitus: rates and risk factors from initial GDM and one abnormal GTT value. Diabetes Res. Clin. Pract. (2006) 71:75–81. doi: 10.1016/j.diabres.2005.05.011 16005100

[202] Boghossian NS Yeung E Albert PS Mendola P Laughon SK Hinkle SN. Changes in diabetes status between pregnancies and impact on subsequent newborn outcomes. Am. J. Obstet Gynecol. (2014) 210:431.e1–14. doi: 10.1016/j.ajog.2013.12.026 PMC4011935 24361790

[203] Berger H Crane J Farine D Armson A de la Ronde S Keenan-Lindsay L. Screening for gestational diabetes mellitus. J. Obstet Gynaecol Can. (2002) 24:894–912. doi: 10.1016/S1701-2163(16)31047-7 12417905

[204] Moses RG. The recurrence rate of gestational diabetes in subsequent pregnancies. Diabetes Care. (1996) 19:1348–50. doi: 10.2337/diacare.19.12.1348 8941462

[205] MacNeill S Dodds L Hamilton DC Armson BA VandenHof M. Rates and risk factors for recurrence of gestational diabetes. Diabetes Care. (2001) 24:659–62. doi: 10.2337/diacare.24.4.659 11315827

[206] Schwartz N Nachum Z Green MS. The prevalence of gestational diabetes mellitus recurrence–effect of ethnicity and parity: a metaanalysis. Am. J. Obstet Gynecol. (2015) 213:310–7. doi: 10.1016/j.ajog.2015.03.011 25757637

[207] Schwartz N Nachum Z Green MS. Risk factors of gestational diabetes mellitus recurrence: a meta-analysis. Endocrine. (2016) 53:662–71. doi: 10.1007/s12020-016-0922-9 27000082

[208] Schwartz N Green MS Yefet E Nachum Z. Modifiable risk factors for gestational diabetes recurrence. Endocrine. (2016) 54:714–22. doi: 10.1007/s12020-016-1087-2 27601018

[209] Schwartz N Green MS Yefet E Nachum Z. Postprandial glycemic control during gestational diabetes pregnancy predicts the risk of recurrence. Sci. Rep. (2018) 8:6350. doi: 10.1038/s41598-018-24314-1 29679039 PMC5910411

[210] Yefet E Schwartz N Nachum Z. Characteristics of pregnancy with gestational diabetes mellitus and the consecutive pregnancy as predictors for future diabetes mellitus type 2. Diabetes Res. Clin. Pract. (2022) 186:109826. doi: 10.1016/j.diabres.2022.109826 35283264

[211] Gabbe’s Obstetrics: Normal and Problem Pregnancies. 8th Edition. Alpharetta, GA 30005, USA: Elsevier (2020).

[212] Yefet E Bejerano A Iskander R Zilberman Kimhi T Nachum Z. The association between gestational diabetes mellitus and infections in pregnancy-systematic review and meta-analysis. Microorganisms. (2023) 11:1956. doi: 10.3390/microorganisms11081956 37630515 PMC10458027

[213] Nachum Z Amir M Shalev E. Long-term implications of gestational diabetes mellitus for the mother. Isr. J. Obstet Gynecol. (2001) 12:142–7.

[214] Noctor E Crowe C Carmody LA Kirwan B O’Dea A Glynn LG. ATLANTIC-DIP: prevalence of metabolic syndrome and insulin resistance in women with previous gestational diabetes mellitus by International Association of Diabetes in Pregnancy Study Groups criteria. Acta Diabetol. (2015) 52:153–60. doi: 10.1007/s00592-014-0621-z 25002067

[215] Catalano PM Vargo KM Bernstein IM Amini SB. Incidence and risk factors associated with abnormal postpartum glucose tolerance in women with gestational diabetes. Am. J. Obstet Gynecol. (1991) 165:914–9. doi: 10.1016/0002-9378(91)90438-w 1951553

[216] O’Sullivan JB. Body weight and subsequent diabetes mellitus. JAMA. (1982) 248:949–52. doi: 10.1001/jama.1982.03330080031024 7097963

[217] Herath H Herath R Wickremasinghe R. Gestational diabetes mellitus and risk of type 2 diabetes 10 years after the index pregnancy in Sri Lankan women-A community based retrospective cohort study. PloS One. (2017) 12:e0179647. doi: 10.1371/journal.pone.0179647 28644881 PMC5482451

[218] Feig DS Zinman B Wang X Hux JE. Risk of development of diabetes mellitus after diagnosis of gestational diabetes. CMAJ. (2008) 179:229–34. doi: 10.1503/cmaj.080012 PMC2474881 18663202

[219] Greenberg LR Moore TR Murphy H. Gestational diabetes mellitus: antenatal variables as predictors of postpartum glucose intolerance. Obstet Gynecol. (1995) 86:97–101. doi: 10.1016/0029-7844(95)00103-X 7784031

[220] Skajaa GO Fuglsang J Knorr S Møller N Ovesen P Kampmann U. Changes in insulin sensitivity and insulin secretion during pregnancy and post partum in women with gestational diabetes. BMJ Open Diabetes Res. Care. (2020) 8:e001728. doi: 10.1136/bmjdrc-2020-001728 PMC7594208 33115822

[221] Hiersch L Shah BR Berger H Geary M McDonald SD Murray-Davis B. Oral glucose tolerance test results in pregnancy can be used to individualize the risk of future maternal type 2 diabetes mellitus in women with gestational diabetes mellitus. Diabetes Care. (2021) 44:1860–7. doi: 10.2337/dc21-0659 34131049

[222] Yefet E Schwartz N Sliman B Nachum Z. One elevated oral glucose tolerance test value in pregnancy increases the risk for future diabetes mellitus type 2. Arch. Gynecol Obstet. (2021) 303:933–41. doi: 10.1007/s00404-020-05827-5 33057771

[223] Varner MW Rice MM Landon MB Casey BM Reddy UM Wapner RJ. Pregnancies after the diagnosis of mild gestational diabetes mellitus and risk of cardiometabolic disorders. Obstet Gynecol. (2017) 129:273–80. doi: 10.1097/AOG.0000000000001863 PMC5352568 28079773

[224] Kaiser K Nielsen MF Kallfa E Dubietyte G Lauszus FF. Metabolic syndrome in women with previous gestational diabetes. Sci. Rep. (2021) 11:11558. doi: 10.1038/s41598-021-90832-0 34078945 PMC8172609

[225] Yefet E Schwartz N Sliman B Ishay A Nachum Z. Good glycemic control of gestational diabetes mellitus is associated with the attenuation of future maternal cardiovascular risk: a retrospective cohort study. Cardiovasc. Diabetol. (2019) 18:75. doi: 10.1186/s12933-019-0881-6 31167664 PMC6549350

[226] Fadl H Magnuson A Östlund I Montgomery S Hanson U Schwarcz E. Gestational diabetes mellitus and later cardiovascular disease: a Swedish population based case-control study. BJOG. (2014) 121:1530–6. doi: 10.1111/1471-0528.12754 PMC4232923 24762194

[227] Lekva T Michelsen AE Aukrust P Henriksen T Bollerslev J Ueland T. Leptin and adiponectin as predictors of cardiovascular risk after gestational diabetes mellitus. Cardiovasc. Diabetol. (2017) 16:5. doi: 10.1186/s12933-016-0492-4 28068986 PMC5223461

[228] Kramer CK Campbell S Retnakaran R. Gestational diabetes and the risk of cardiovascular disease in women: a systematic review and meta-analysis. Diabetologia. (2019) 62:905–14. doi: 10.1007/s00125-019-4840-2 30843102

[229] Kalhan SC Savin SM Adam PA. Attenuated glucose production rate in newborn infants of insulin-dependent diabetic mothers. N Engl. J. Med. (1977) 296:375–6. doi: 10.1056/NEJM197702172960706 834200

[230] Riskin A Itzchaki O Bader D Iofe A Toropine A Riskin-Mashiah S. Perinatal outcomes in infants of mothers with diabetes in pregnancy. Isr. Med. Assoc. J. (2020) 22:569–75. 33236556

[231] Demarini S Mimouni F Tsang RC Khoury J Hertzberg V. Impact of metabolic control of diabetes during pregnancy on neonatal hypocalcemia: a randomized study. Obstet Gynecol. (1994) 83:918. doi: 10.1097/00006250-199406000-00003 8190431

[232] Miouni F Miodovnik M Whitsett JA. Respiratory distress syndrome in infants of diabetic mothers in the 1980s: no direct adverse effect of maternal diabetes with modern management. Obstet Gynecol. (1987) 69:191–5. 3808504

[233] Topcuoglu S Karatekin G Yavuz T Arman D Kaya A Gursoy T. The relationship between the oxidative stress and the cardiac hypertrophy in infants of diabetic mothers. Diabetes Res. Clin. Pract. 109:104–9. doi: 10.1016/j.diabres.2015.04.022 25934526

[234] Ullmo S Vial Y Di Bernardo S Roth-Kleiner M Mivelaz Y Sekarski N. Pathologic ventricular hypertrophy in the offspring of diabetic mothers: A retrospective study. Eur. Heart J. (2007) 28:1319–25. doi: 10.1093/eurheartj/ehl416 17158827

[235] Leybovitz-Haleluya N Wainstock T Landau D Sheiner E. Maternal gestational diabetes mellitus and the risk of subsequent pediatric cardiovascular diseases of the offspring: a population-based cohort study with up to 18 years of follow up. Acta Diabetol. (2018) 55:1037–42. doi: 10.1007/s00592-018-1176-1 29936651

[236] Werner EF Romano ME Rouse DJ Sandoval G Gyamfi-Bannerman C Blackwell SC. Association of gestational diabetes mellitus with neonatal respiratory morbidity. Obstetrics gynecology. (2019) 133:349–53. doi: 10.1097/AOG.0000000000003053 PMC6357223 30633135

[237] Dabelea D Mayer-Davis EJ Lamichhane AP D’Agostino RB Jr Liese AD Vehik KS. Association of intrauterine exposure to maternal diabetes and obesity with type 2 diabetes in youth: the SEARCH case-control study. Diabetes Care. (2008) 31:1422–6. doi: 10.2337/dc07-2417 PMC2453655 18375420

[238] Holder T Giannini C Santoro N Pierpont B Shaw M Duran E. A low disposition index in adolescent offspring of mothers with gestational diabetes: a risk marker for the development of impaired glucose tolerance in youth. Diabetologia. (2014) 57:2413–20. doi: 10.1007/s00125-014-3345-2 25168408

[239] Abokaf H Shoham-Vardi I Sergienko R Landau D Sheiner E. *In utero* exposure to gestational diabetesmellitus and long term endocrine morbidity of the offspring. Diabetes Res. Clin. Pract. (2018) 144:231–5. doi: 10.1016/j.diabres.2018.09.003 30213770

[240] Sweeting A Wong J Murphy HR Ross. GP. A clinical update on Gestational Diabetes Mellitus. Endocr. Rev. (2022) 43:763–93. doi: 10.1210/endrev/bnac003 PMC9512153 35041752

[241] Adane AA Mishra GD Tooth LR. Diabetes in pregnancy and childhood cognitive development: A systematic review. Pediatrics. (2016) 137:e20154234. doi: 10.1542/peds.2015-4234 27244820

[242] Nahum Sacks K Friger M Shoham-Vardi I Abokaf H Spiegel E Sergienko R. Prenatal exposure to gestational diabetes mellitus as an independent risk factor for long-term neuropsychiatric morbidity of the offspring. Am J Obstet Gynecol. (2016) 215:380.e1–7. doi: 10.1016/j.ajog.2016.03.030 27018463

