# Supplementary material for: Gestational diabetes mellitus – more than the eye can see – a warning sign for future maternal health with transgenerational impact
Source: Front Clin Diabetes Healthc. 2025 Apr 1;6:1527076. doi: 10.3389/fcdhc.2025.1527076 (PMC11997571; doi:10.3389/fcdhc.2025.1527076)
Supplement: Supplementary file 1 [file Table1.docx]

Supplementary table 1 – diagnostic testing and criteria for gestational diabetes according to different societies and guidelines

|  | Routine/risk stratification | Early testing | Routine testing | Diagnostic criteria for GDM |
| --- | --- | --- | --- | --- |
| ADIPS 2014 | Routine | Depending on risk factors.  Methods of testing* -Ideally POGTT or HbA1C. | One step strategy  A75g OGTT at 24-28 weeks' gestation. | Any of the following:  (a) Fasting plasma glucose 5.1–6.9 mmol/L (92−125 mg/dL).  (b) 1-h post 75 g oral glucose load ≥ 10.0 mmol/L (180 mg/dL). **  (c) 2-h post 75 g oral glucose load 8.5–11.0 mmol/L (153−199 mg/dL). |
| RCOG (published 2015, updated 2020) | Risk factors | For women with GDM in prior pregnancy.  Methods- 1-early self-monitoring of blood glucose  2-75g 2 hours OGTT. | One step strategy  A 75g 2-hour OGTT at 24 – 28 weeks. | Any of the following:  (a) Fasting plasma glucose level ≥ 5.6 mmol/L (101 mg/dL).  (b) 2-h post 75 g oral glucose load ≥ 7.8 mmol/L (140 mg/dL). |
| ADA 2022 | Routine |  | The “one-step strategy” 75-g OGTT  OR  The “two-step” approach with a 50-g (non-fasting) GCT followed by a 100-g (fasting) OGTT for those who screen positive. | One step approach, any of the following:  (a) Fasting plasma glucose level ≥ 5.1 mmol/L (92 mg/dL)  (b) 1-h post 75 g oral glucose load ≥ 10.0 mmol/L (180 mg/dL)  (c) 2-h post 75 g oral glucose load ≥ 8.5 mmol/L (153 mg/dL)  Two step approach:  (1) 1-hr post 50g oral glucose load ≥ 7.2, 7.5, or 7.8 mmol/L (130, 135, 140 mg/dL, respectively).  Proceed to step 2.  (2) At least two of the following post 100-g OGTT (Carpenter-Coustan criteria)-  Fasting: 95 mg/dL (5.3 mmol/L)  1 h≥ 10.0 mmol/L (180 mg/dL)  2 h≥ 8.6 mmol/L (155 mg/dL)  3 h≥ 7.8 mmol/L (140 mg/dL). |
| FIGO 2015 | Routine |  | One step strategy  A 75g OGTT at 24-28 weeks gestation. | One or more of the following:  (a) Fasting plasma glucose 5.1–6.9 mmol/L (92−125 mg/dL).  (b) 1-h post 75 g oral glucose load ≥ 10.0 mmol/L (180 mg/dL).  (c) 2-h post 75 g oral glucose load 8.5–11.0 mmol/L (153−199 mg/dL). |
| SOGC 2019 | Routine | Depending on risk factors, screening or testing should be offered during the first half of the pregnancy. | Preferred: two step strategy, with a 50-g (non-fasting) GCT followed by a 2-hour 75g OGTT.  Alternative: – one step strategy 2-hour 75g OGTT. | Two step approach:  (1) 1-hr post 50g oral glucose load is equal to 7.8 – 11.0 mmol/L (140 – 190 mg/dL) then proceed to step 2.  If the value of the GCT ≥ 11.1 mmol/L (200 mg/dL), GDM is diagnosed.  (2) One or more of the following:  - Fasting plasma glucose ≥ 5.3 mmol/L (95 mg/dL)  - 1-h post 75 g oral glucose load ≥ 10.6 mmol/L (191 mg/dL)  - 2-h post 75 g oral glucose load ≥ 9.0 mmol/L (162 mg/dL).  One step approach:  One or more of the following-  (a) Fasting plasma glucose ≥ 5.1 mmol/L (92 mg/dL)  (b) 1-h post 75 g oral glucose load ≥ 10.0 mmol/L (180 mg/dL)  (c) 2-h post 75 g oral glucose load 8.5 mmol/L (153 mg/dL) |
| WHO 2018 | Routine | Depending on risk factors, starting at 1st trimester | One step strategy 2-hour 75g OGTT. | One or more of the following:  (a) Fasting plasma glucose 5.1–6.9 mmol/L (92−125 mg/dL)  (b) 1-h post 75 g oral glucose load ≥ 10.0 mmol/L (180 mg/dL)  (c) 2-h post 75 g oral glucose load 8.5–11.0 mmol/L (153−199 mg/dL). |

ADIPS - Australasian Diabetes in Pregnancy Society; RCOG - Royal College of Obstetricians and Gynaecologists ; ADA – American diabetes association ; FIGO - The International Federation of Gynecology and Obstetrics ; SOGC - The Society of Obstetricians and Gynaecologists of Canada; WHO - World Health Organization; POGTT – pregnancy oral glucose tolerance test; HbA1C - hemoglobin A1C; OGTT - oral glucose tolerance test; GDM - gestational diabetes mellitus; GCT – glucose challenge test.
